# The effect of high protein dosing in critically ill patients: an exploratory, secondary Bayesian analyses of the EFFORT Protein trial

**DOI:** 10.1016/j.bja.2024.08.033

**Published:** 2024-10-24

**Authors:** Ryan W. Haines, Anders Granholm, Zudin Puthucheary, Andrew G. Day, Danielle E. Bear, John R. Prowle, Daren K. Heyland

**Affiliations:** 1 Adult Critical Care Unit, The Royal London Hospital, Barts Health NHS Trust, London, UK; 2 William Harvey Research Institute, Queen Mary University of London, London, UK; 3 Department of Intensive Care, Copenhagen University Hospital–Rigshospitalet, Copenhagen, Denmark; 4 Clinical Evaluation Research Unit, Kingston Health Science Center, Kingston, ON, Canada; 5 Department of Critical Care, Guy's and St. Thomas' NHS Foundation Trust, London, UK; 6 Department of Nutrition and Dietetics, Guy's and St. Thomas' NHS Foundation Trust, London, UK; 7 Department of Renal Medicine and Transplantation, The Royal London Hospital, Barts Health NHS Trust, London, UK; 8 Department of Critical Care Medicine, Queen's University, Kingston, ON, Canada

**Keywords:** Bayesian analysis, heterogeneity of treatment effects, intensive care, multi-organ failure, protein

## Abstract

**Background:**

The EFFORT Protein trial assessed the effect of high *vs* usual dosing of protein in adult ICU patients with organ failure. This study provides a probabilistic interpretation and evaluates heterogeneity in treatment effects (HTE).

**Methods:**

We analysed 60-day all-cause mortality and time to discharge alive from hospital using Bayesian models with weakly informative priors. HTE on mortality was assessed according to disease severity (Sequential Organ Failure Assessment [SOFA] score), acute kidney injury, and serum creatinine values at baseline.

**Results:**

The absolute difference in mortality was 2.5% points (95% credible interval –6.9 to 12.4), with a 72% posterior probability of harm associated with high protein treatment. For time to discharge alive from hospital, the hazard ratio was 0.91 (95% credible interval 0.80 to 1.04) with a 92% probability of harm for the high-dose protein group compared with the usual-dose protein group. There were 97% and 95% probabilities of positive interactions between the high protein intervention and serum creatinine and SOFA score at randomisation, respectively. Specifically, there was a potentially relatively higher mortality of high protein doses with higher baseline serum creatinine or SOFA scores.

**Conclusions:**

We found moderate to high probabilities of harm with high protein doses compared with usual protein in ICU patients for the primary and secondary outcomes. We found suggestions of *heterogeneity in treatment effects* with worse outcomes in participants randomised to high protein doses with renal dysfunction or acute kidney injury and greater illness severity at baseline.

**Editor's key points**

• This Bayesian analysis of the EFFORT Protein trial provides supporting evidence that high protein compared with usual protein dosing in critically ill patients might cause harm, defined as 60-day mortality and time to discharge alive from hospital at 60 days post-randomisation.
• A heterogeneity of treatment effects (HTE) approach was used to assess the effect of disease severity, renal impairment, and BMI on the outcomes.
• HTE models demonstrate a high probability of worse outcomes in patients with high baseline SOFA scores and those with renal impairment, but a low probability of an effect attributed to BMI.
• The effects of these factors on the outcomes were assessed as highly credible using the ICEMAN instrument.

Establishing the optimal dose of protein has been identified as a research priority in the field of critical care nutrition to inform clinical practice.^1^ Although protein is a fundamental requirement for stimulation of protein synthesis and cellular function and survival, the current evidence remains insufficient to recommend an optimum dose in critical illness.^2^

In an 85-centre study of 1301 critically ill patients, the EFFORT Protein trial randomised participants to target 2.2 g kg^−1^ day^−1^ or more of protein *vs* 1.2 g kg^−1^ day^−1^ or less for 28 days.^3^ The primary, frequentist analysis found no statistically significant difference in mortality (relative risk 1.08, 95% confidence interval 0.92–1.26) or time to discharge alive from hospital (hazard ratio [HR] 0.91, 95% confidence interval 0.77–1.07). Furthermore, harm from high protein was estimated in predefined subgroups of participants with acute kidney injury (AKI) and high organ failure scores.

We conducted this exploratory, secondary Bayesian analysis to provide a probabilistic interpretation of the primary and secondary outcomes in the EFFORT Protein trial. A Bayesian analysis can ‘augment’^4^ interpretation by providing clinicians and researchers with direct probabilities and a way to quantify uncertainties by providing an interpretable interval where the true parameter value falls.^5^, ^6^, ^7^, ^8^, ^9^ Furthermore, we examined the suggested heterogeneity of treatment effects (HTE) reported in the EFFORT Protein trial.

Conventional HTE analyses defining subgroups by dichotomising variables have limitations, including the increased risk of chance findings as a result of multiple testing.^10^^,^^11^ A more detailed analyses of HTE such as avoiding dichotomisation may provide clearer probabilities of estimates of interaction effects and therefore more useful evidence on how to potentially refine interventions for future clinical trials.^12^

In the original EFFORT Protein trial, the aim was to test the hypothesis that a high dose of protein provided to critically ill patients would improve their clinical outcomes. In this re-analysis, we aim to estimate the probabilities of different effects of high protein on mortality and time to discharge alive from hospital and explore the potential of using a Bayesian framework.

## Methods

### Study design

We performed a secondary Bayesian analysis of the EFFORT Protein trial. This study adheres to the Strengthening the Reporting of Observational Studies in Epidemiology (STROBE) statement (see the Supplementary material).^4^ The analysis was conducted in accordance with the Reporting Of Bayes Used in clinical Studies (ROBUST) guidelines.^13^ An internal protocol and statistical analysis plan was prepared after the results of the EFFORT Protein trial were available, but before the present analyses unless stated, and is included in the Supplementary material.

### EFFORT Protein trial

The EFFORT Protein trial was registered at ClinicalTrials.gov (NCT03160547, May 17, 2017). It was a multicentre, pragmatic, volunteer-driven, registry-based, randomised, open-label, clinical trial comparing a high protein dose (≥2.2 g kg^−1^ day^−1^) to a usual dose (≤1.2 g kg^−1^ day^−1^) prescription in terms of time to discharge alive from hospital and 60-day all-cause mortality. The trial included 1301 adult (≥18 yr) participants with nutritional risk factors and requiring mechanical ventilation for at least 48 h.^3^^,^^14^

The investigator-initiated EFFORT Protein trial protocol was approved by the Research Ethics Committees of Queen's University, Canada, and a central institutional review board (IRB) at Vanderbilt University, Nashville, TN, USA, that granted a waiver of informed consent for sites that acceded to this central IRB.

### COVID pandemic trial adjustments

Because of the strain of the COVID-19 pandemic on critical care services and research prioritisation, the original enrolment goals were changed by the study steering committee on September 15, 2021, to end study enrolment at 1200 participants, as opposed to the originally planned 4000 participants. This reduced the power for the preplanned frequentist analysis, whereby to achieve 80% power, the originally planned reduction of the primary outcome of 60-day mortality from 30% to 26% was now required to be 22.9% (a 24% relative risk reduction).^14^ Consequently, the secondary outcome of time to discharge alive from hospital was changed to the primary outcome owing to the higher power (83%) to detect differences in effect size at the revised sample size.^3^ For our secondary Bayesian analysis, we kept 60-day all-cause mortality as the primary outcome to aid interpretation of effect estimates for this outcome in the Bayesian framework.

### Primary outcome

The primary outcome was all-cause mortality within 60 days after randomisation.

### Secondary outcome

The secondary outcome was time to discharge alive from hospital up to 60 days after randomisation.

### Statistical analyses

All statistical analyses were conducted using R version 4.1.2 (R Core Team, R Foundation for Statistical Computing, Vienna, Austria) and Stan^15^ (RStan version 2.21.0) through the brms R package (version 2.20.3).^16^ We used data from all participants in the modified intention-to-treat population reported in the primary publication of the EFFORT Protein trial (28 participants were excluded because they never received the intervention because of early death, early discharge, or withdrawal of consent).^3^

### Descriptive data

Descriptive data for the full trial cohort and all HTE subgroups (defined below) stratified by treatment group are summarised as medians (interquartile ranges) for numerical data and as counts (percentages) for categorical data. For models examining HTE for creatinine on a continuous scale, we summarised quartile-based groups stratified by treatment group.

### Bayesian analyses

We used Bayesian inference to combine a range of prior probability distributions with the EFFORT Protein trial data to estimate posterior probability distributions of the treatment effect using a similar approach to previous analyses of randomised controlled trials.^4^^,^^17^ Priors of the treatment effect were prespecified in all models before analyses. We present the full posterior distributions graphically and summarise them by their medians (point estimates) and 95% percentile-based credible intervals (CrIs).^18^^,^^19^ To augment the interpretation of the EFFORT Protein trial, we present probabilities of any benefit/harm, clinically important benefit/harm, and no clinically important difference according to predefined thresholds (specified below). Model specifications and details on model diagnostics are presented in the Supplementary material.

### Priors

We used a family of three priors (a neutral sceptical prior, an optimistic prior favouring the high dose, and a pessimistic prior favouring the usual dose), all assuming a normal distribution of the log odds ratio (OR) and with a moderate uncertainty of the effect size. The sceptical prior presented was centred on no difference between treatment groups and allowed for a range of plausible effect sizes (Supplementary Fig. S1).

### Primary and secondary outcomes

We used hierarchical Bayesian logistic regression models adjusted for the stratification variable site (random effect) to analyse the binary outcome 60-day mortality with results presented as conditional risk ratios (RRs) and risk differences (RDs), and secondarily as conditional ORs, in each group (with the largest site, i.e. the Mexico Hospital Civil Fray Antonio Alcalde site, being the reference). We considered an absolute RD of ≥2.0% points as the minimally clinically important difference, consistent with thresholds used in similar studies.^6^, ^7^, ^8^ We analysed time to discharge alive from hospital using Bayesian Cox regression in *brms* adjusted for the stratification variable site (fixed effect) and a similar family of priors (sceptical, optimistic, and pessimistic; Supplementary Fig. S2). We censored deaths at the end of the 60-day follow-up period to approximate the Fine–Gray approach, with death as a competing risk precluding discharge.^20^ Subdistribution HRs of time to discharge alive from hospital are presented.

### Heterogeneity of treatment effects

We evaluated the presence of HTE for the primary outcome of 60-day mortality using several approaches. First, to replicate the reported interactions suggesting HTE in the EFFORT Protein trial frequentist analyses, we used hierarchical Bayesian logistic regression models with interactions for baseline groups, including AKI (yes/no), and illness severity groups (Sequential Organ Failure Assessment [SOFA] <9 *vs* ≥9).^3^ Second, to extend the frequentist analyses, we used hierarchical Bayesian logistic regression with interaction models to assess HTE with creatinine (highest creatinine at randomisation) and SOFA on a continuous scale. Third, we investigated the potential of HTE using BMI-defined subgroups (BMI ≤30 *vs* >30) in a *post hoc* analysis based on the recent publication of a secondary analysis of the EFFORT Protein trial.^21^ In addition, we used a hierarchical Bayesian logistic regression with an interaction model to assess HTE with BMI on a continuous scale. Our primary goal was to assess which participant characteristics were associated with alterations in treatment efficacy. In HTE models, we used the same sceptical prior for the treatment effect. For interaction and subgroup effects, we used weakly informative priors that allowed the likelihood to dominate the posterior. We used the Instrument to assess the Credibility of Effect Modification Analyses (ICEMAN) checklist.^22^

### Missing data handling

All analyses were complete cases only, with numbers of participants excluded in adjusted models reported with descriptors. In addition, the primary and secondary outcome data were missing in four participants. All analyses were adjusted for the stratification variable trial site.

## Results

A total of 1301 participants (97.9%) were included (645 assigned to the high-dose protein group and 656 to the usual-dose protein group). Baseline characteristics were similar across the two treatment groups (Supplementary Table S1). All model diagnostics were considered acceptable (details in the Supplementary material).

### Primary outcome

**Fig 1:**
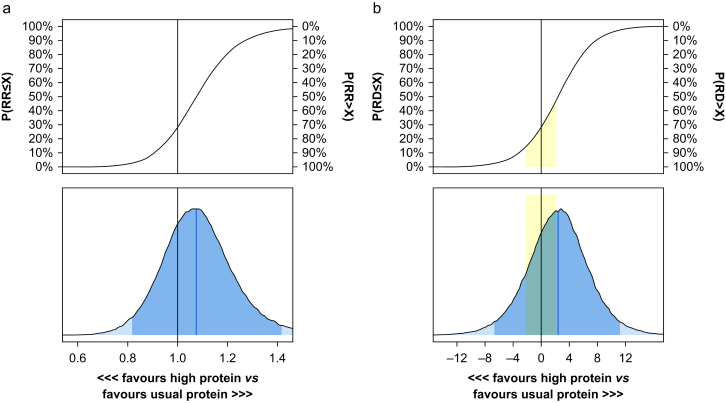
Posterior probability distributions for 60-day mortality. Analyses using weakly informative priors adjusted for trial site (random effect). The black vertical lines represent no difference. (top) Cumulative posterior distributions of effect sizes. (bottom) Corresponding posterior density plots with medians (blue vertical lines) and percentile-based 95% credible intervals (CrI, darker blue areas). (a) Risk ratio (RR) with a median of 1.08 (95% CrI 0.82–1.44). (b) Risk difference (RD) with a median of 2.5% points (95% CrI –6.9 to 12.4). The yellow area demarks effect sizes smaller than the predefined minimally clinically important effect. X denotes various treatment effect sizes on the horizontal axis with the corresponding probabilities of RR ≤ X or RD ≤ X values on the left y-axis and RR > X or RD > X on the right y-axis.

For 60-day all-cause mortality with a weakly informative sceptical prior, the RD was 2.5% (95% CrI –6.9 to 12.4), corresponding to an RR of 1.08 (0.82–1.44). The probability of any harm (i.e. an RD >0.0% points) with higher protein doses was 72%, and the probability of clinically important harm was 54%. The probability of no clinically important difference (i.e. an RD > –2.0% points and an RD <2.0% points) was 31%. The full posterior probability distributions are illustrated in Figure 1.

In the sensitivity analyses using an optimistic prior, the probability of any harm from higher protein doses was 57%, and the probability of clinically important harm was 37% (Supplementary Figs S3 and S4). In the sensitivity analyses using a pessimistic prior, the probability of any harm from higher protein doses was 78%, and the probability of clinically important harm was 61% (Supplementary Figs S5 and S6). The estimated treatment effects and probabilities of select effect sizes for all sets of priors are presented in Table 1. The full posterior probability distributions of the sensitivity analyses are presented in the Supplementary material.

**Table 1:** Results of Bayesian analysis of 60-day mortality from EFFORT Protein trial. Priors were set following a statistical analysis plan, which used the suggested principles outlined by Zampieri and colleagues,^4^ using sceptical, optimistic, and pessimistic priors of moderate strength at (a) *N*(0, 0.355), (b) *N*(–0.198, 0.195), and (c) *N*(0.198, 0.195), respectively. Priors are shown in Supplementary Figure S1. All analyses were conducted in the modified intention-to-treat population after the exclusion of four participants because of missing primary outcome data (*n*=1297). All analyses were adjusted for the trial site (random effect). The effect estimates are reported as conditional treatment effects with median posterior values as point estimates and percentile-based 95% CrIs. All definitions of thresholds for clinically important differences are listed in the Methods section. Any benefit is the probability of an RD <0.0% (RR <1.00). Similarly, any harm is the probability of an RD >0.0% points (RR >1.00). Clinically important benefit is defined as the probability of an RD ≤−2.0% points, and clinically important harm is the probability of an RD ≥2.0% points. No clinically important difference is the probability of an RD >−2.0% and an RD <2.0%. Crl, credible interval; RD, risk difference; RR, risk ratio.

	High protein group, probability (95% CrI) (%)	Usual protein group, probability (95% CrI) (%)	Relative difference, RR (95% CrI)	Absolute difference, RD (95% CrI) (%)	Probability of any harm, Pr(RR >1)	Probability of clinically important benefit, Pr(RD ≤–2%) (%)	Probability of clinically important harm, Pr(RD ≥ 2%) (%)	Probability of no clinically important difference, Pr(RD >–2.0% and RD <2.0%) (%)
Primary analysis using a sceptical prior	34.5 (28.5–42.4)	32.2 (26.2–39.3)	1.08 (0.82–1.44)	2.5 (–6.9 to 12.4)	72	15	54	31
Sensitivity analysis using an optimistic prior	33.7 (27.9–41.0)	32.9 (27.1–40.0)	1.02 (0.79–1.35)	0.8 (–8.3 to 10.3)	57	25	37	38
Sensitivity analysis using a pessimistic prior	34.9 (28.5–42.1)	31.7 (25.8–38.6)	1.10 (0.83–1.45)	3.1 (–6.0 to 12.3)	78	11	61	28

### Secondary outcomes

**Fig 2:**
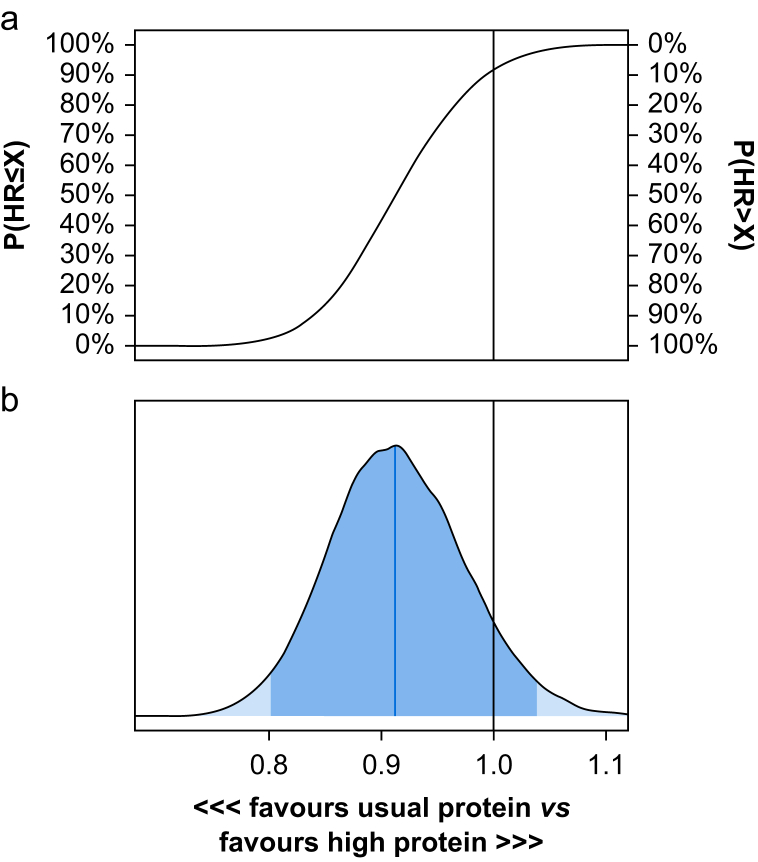
Posterior probability distribution for time to discharge alive from hospital. Models used weakly informative priors adjusted for the trial site (fixed effect for 33 sites; see the Supplementary material for additional details). (a) Cumulative posterior distributions of hazard ratios. (b) Corresponding posterior density plot with median (blue vertical line) and percentile-based 95% credible interval (CrI, darker blue area). Sceptical prior (*N*(1, 0.3)), hazard ratio (HR) with a median of 0.91 (95% CrI 0.80–1.04). X denotes various treatment effect sizes on the horizontal axis with the corresponding probabilities of HR ≤ X values on the left y-axis and HR > X on the right y-axis. HR >1 indicates an increased hazard of discharge from hospital alive.

For time to discharge alive from hospital, the HR was 0.91 (95% CrI 0.80 to 1.04), with a 92% probability of HR <1 (harm) for the high-dose protein group compared with the usual-dose protein group in models with a weakly informative, sceptical prior (Fig. 2). In sensitivity analyses with optimistic and pessimistic priors, there was an 89% and 94% probability of HR <1, respectively (Supplementary Figs S7 and S8 and Table S2).

### Heterogeneity of treatment effects

**Fig 3:**
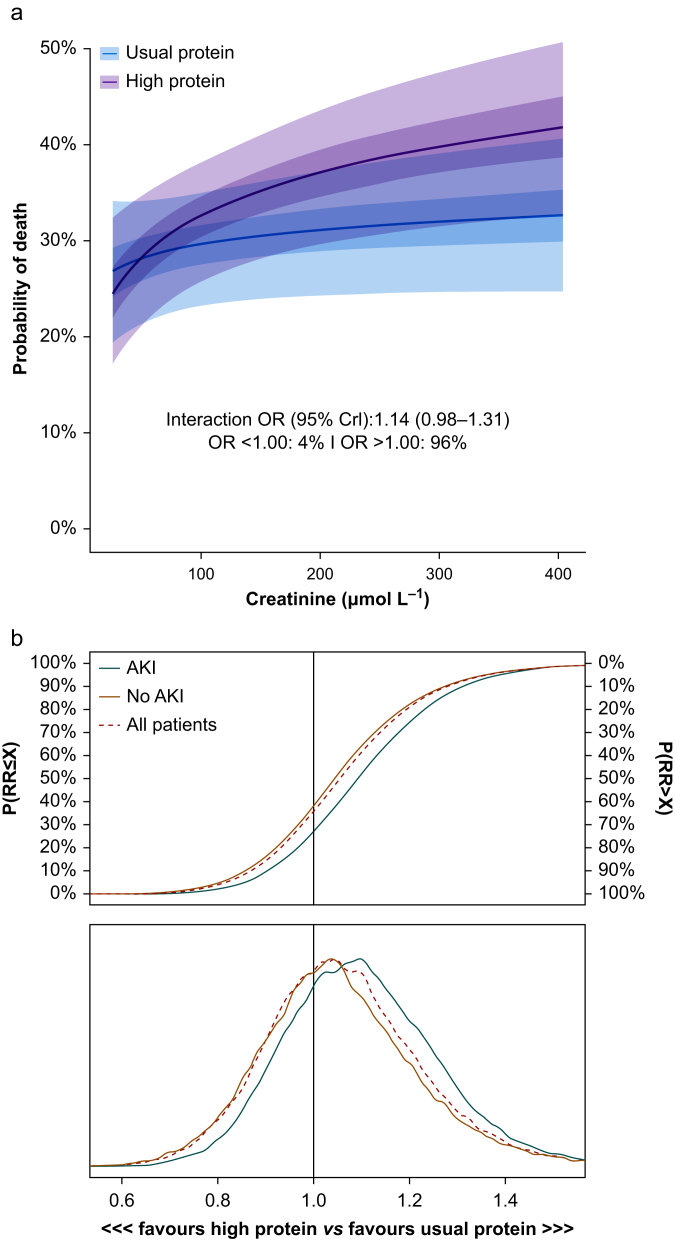
Heterogeneity of treatment effect analyses according to baseline serum creatinine and presence of acute kidney injury using weakly informative priors. Analysis of heterogeneity of treatment effects for 60-day all-cause mortality using weakly informative priors. (a) Conditional effects of the interaction between treatment allocation and baseline serum creatinine TS: (highest creatinine in 24 h before randomisation). The plot displays the estimated mortality risk on the vertical axis and serum creatinine values on the horizontal axis. The conditional odds ratio (OR) of the interaction of treatment and a doubling of serum creatinine at baseline (or 1 unit increase in log2 serum creatinine) is 1.14 (95% credible interval 0.98–1.31), and there is a 96% probability that mortality in the high protein group increases with a doubling of creatinine. Ninety participants were missing baseline creatinine values and were thus excluded. (b) The cumulative posterior probability distributions with corresponding posterior density plots of the conditional risk ratios of 60-day all-cause mortality in the full sample and by acute kidney injury (AKI) groups (presence of AKI [KDIGO stage 1 or above] at randomisation).

Baseline characteristics according to the HTE subgroups and quartiles of baseline creatinine values (analysed without categorisation) are presented in Supplementary Tables S3–S6. There was a 97% probability of positive interaction between the high protein intervention and baseline serum creatinine at randomisation in the continuous HTE analysis on mortality (i.e. a potential negative impact of higher protein appeared to worsen with increasing baseline serum creatinine measurements; Fig. 3). In the AKI subgroup-based HTE analysis, the posterior distribution for the AKI group at randomisation favoured the usual protein group corresponding to an RD of 4.2% points (95% CrI –7.8%–15.7%) in 60-day all-cause mortality (Table 2). In the no AKI group at randomisation, we found an RD in the same direction but at a smaller magnitude of 1.5% points (95% CrI –7.37%–10.5%). There was an 80% probability of a negative interaction between the high protein intervention and baseline BMI at randomisation in the continuous HTE analysis on mortality and minimal differences in 60-day all-cause mortality between BMI subgroups (Supplementary Fig. S9 and Table 2).

**Table 2:** Summarised effect measures for all-cause mortality using weakly informative (sceptical) prior. All analyses were conducted in the modified intention-to-treat population after the exclusion of four participants in the analyses because of missing primary outcome data (*n*=1297). Ninety participants were missing baseline creatinine values. AKI, acute kidney injury; CrI, credible interval; RD, risk difference (>0.0% points favours the usual protein group); RR, risk ratio (>1.00 favours the usual protein group); SOFA, Sequential Organ Failure Assessment.

Group	*N*	High protein group, probability (95% CrI) (%)	Usual protein group, probability (95% CrI) (%)	Relative difference, RR (95% CrI)	Absolute difference, RD (95% CrI) (%)
All patients	1297	34.5 (28.5–42.4)	32.2 (26.2–39.3)	1.08 (0.82–1.44)	2.5 (–6.9 to 12.4)
AKI					
Yes	309	44.8 (36.6–53.4)	40.6 (32.7–49.2)	1.10 (0.83–1.45)	4.2 (–7.8 to 15.7)
No	988	30.8 (24.7–38.0)	29.2 (23.5–35.8)	1.05 (0.78–1.42)	1.5% (–7.37 to 10.5)
SOFA score					
≥9	671	39.2 (32.1–48.2)	35.8 (28.7–45.0)	1.09 (0.82–1.48)	3.4 (–8.1 to 14.7)
<9	626	29.5 (22.8–37.3)	28.2 (21.7–36.0)	1.05 (0.74–1.49)	1.4 (–8.7 to 11.5)
BMI (kg m^−2^)					
>30	878	30.8 (24.2–39.2)	28.4 (22.0–36.1)	1.08 (0.78–1.52)	2.3 (–7.6 to 12.1)
≤30	419	36.2 (29.6–44.1)	33.9 (27.7–41.7)	1.07 (0.80–1.41)	2.4 (–7.9 to 12.1)

**Fig 4:**
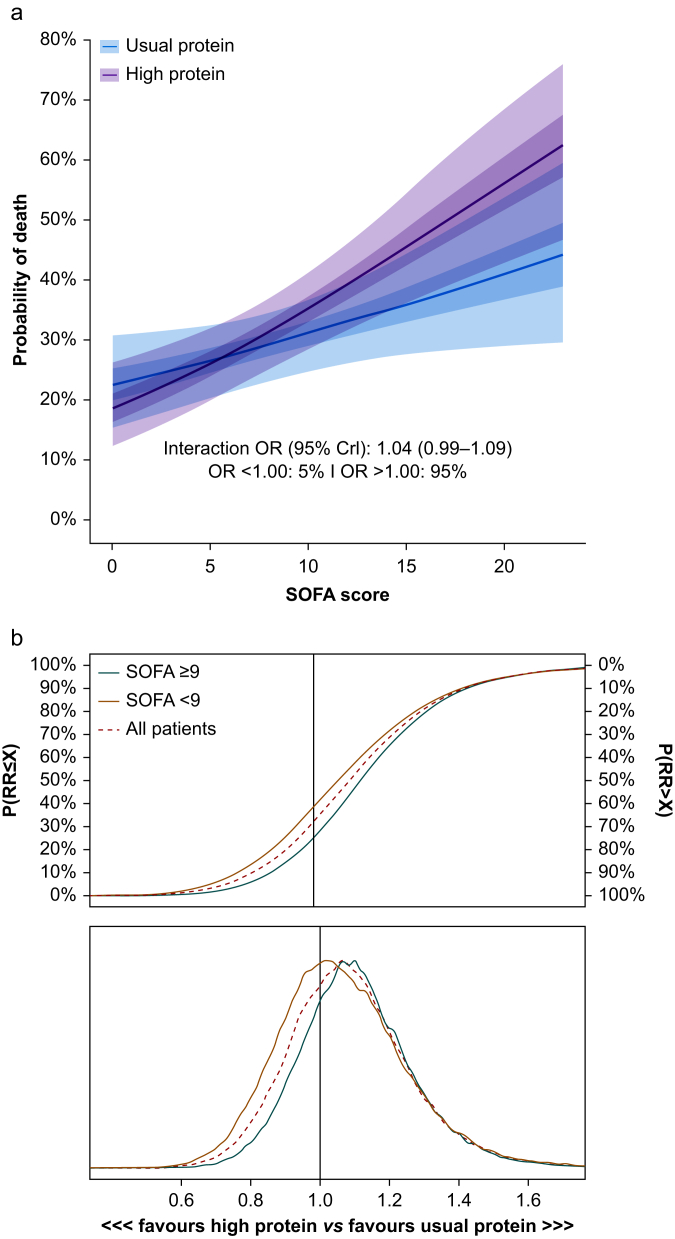
Heterogeneity of treatment effect analyses according to baseline illness severity. Analysis of heterogeneity of treatment effects for 60-day all-cause mortality using weakly informative priors. (a) Conditional effects of the interaction between treatment allocation and baseline Sequential Organ Failure Assessment (SOFA) score (at randomisation). The plot displays the estimated mortality risk on the vertical axis and SOFA score on the horizontal axis. The conditional odds ratio of the interaction of treatment and SOFA score is 1.04 (95% credible interval [CrI] 0.99–1.09), and there is a 95% probability that mortality in the high protein group increases with an increase in SOFA score. (b) The cumulative posterior probability distributions with corresponding posterior density plots of the conditional risk ratios of 60-day all-cause mortality in the full sample and by SOFA groups (SOFA <9 and SOFA ≥9).

In the SOFA HTE analyses, there was a 95% probability of positive interaction between the high protein intervention and baseline SOFA score modelled on a continuous scale on mortality (i.e. a greater increase in mortality for participants randomised to the high protein intervention with higher baseline SOFA scores compared with the usual protein group; Fig. 4). In the SOFA subgroup-based HTE analysis, the posterior distribution for the high SOFA (≥9) group at randomisation favoured the usual protein group corresponding to an RD of 3.4% points (95% CrI: –8.11%–14.7%) in 60-day all-cause mortality (Table 2). In the low SOFA (<9) group at randomisation, we found an RD in the same direction but at a smaller magnitude of 1.5% points (–7.37%–10.5%).

According to the ICEMAN criteria, there was high credibility (very likely effect modification) for SOFA and AKI HTE analyses and very low credibility (minimal to no support for effect modification) for the BMI HTE analyses (Supplementary material).

## Discussion

In this secondary Bayesian analysis of the EFFORT Protein trial, we found posterior probabilities of harm with the high protein intervention compared with usual protein: 72% for mortality and 92% for time to discharge alive from hospital. Furthermore, there was a suggestion that both worse renal dysfunction or AKI and increased illness severity at baseline interacted with the higher protein intervention, increasing the probability of worse outcomes.

Our conclusions from this Bayesian analysis, given in terms of probabilities that harm exists, offer important nuances to the interpretation compared with the primary frequentist analysis and several secondary analyses.^21^^,^^23^, ^24^, ^25^ Using sceptical priors, there was a moderate probability that high protein resulted in harm, which increased to a high probability of harm on time to discharge alive from hospital, the primary outcome of EFFORT Protein. Although the probability of clinically important harm (≥2% increase in absolute risk) was 54%, moderate to high probabilities of any harm when considering both 60-day all-cause mortality and time to discharge alive from hospital suggest a more reasonable basis to inform against the use of higher protein. The estimate of the probability of harm from protein exceeding benefit was evident across different prior beliefs of the treatment effect. Enteral nutrition is a ubiquitous intervention in critically ill patients, and therefore, similar to oxygen^26^ or i.v. fluids,^27^ small differences in effect on patient outcomes potentially have important global public health implications.

These analyses highlight the potential for an interaction between AKI and higher protein doses resulting in additional harm. These findings are similar to the predefined frequentist subgroup analysis, which reported a relative risk of 1.4 (95% confidence interval 1.1–1.8) to participants with AKI randomised to higher protein doses,^3^ and align with the findings of a subsequent *post hoc* frequentist analysis of AKI subgroups.^23^ We further investigated HTE in models using baseline serum creatinine values on a continuous scale, where there was potentially additional harm to participants randomised to high protein doses with increased serum creatinine. Combined, the Bayesian HTE models reinforce the potential for harm of protein for patients with worsened renal function. Similarly, the high probability of an interaction between increasing illness severity at baseline and protein dose either across SOFA subgroups or as a continuous value supports further investigation. Additional HTE analyses of BMI subgroups (BMI >30 *vs* ≤30) and baseline BMI on a continuous scale demonstrated limited evidence for potential interaction and HTE, in line with frequentist *post hoc* analyses.^21^

### Strengths and limitations

The strengths of this study include those of the EFFORT Protein international randomised trial and use of a protocol based on consensus guidelines on Bayesian studies in critical care.^4^ There are several limitations to consider. First, the analysis plan was not prespecified before the result of the EFFORT Protein trial and is therefore at risk of being influenced by knowledge of the results of the primary report and several *post hoc* secondary analyses.^21^^,^^23^, ^24^, ^25^ However, our primary aim was to reframe the primary analysis using a probabilistic Bayesian approach rather than assess new hypotheses regarding the observed effects of higher protein dosing. This was demonstrated using neutral priors and analytical choices that matched the primary analysis. Second, our thresholds for clinically important benefit and harm were chosen based on previous publications,^8^ and other reasonable thresholds could have easily been presented. As the complete posteriors are presented, the probabilities according to other thresholds can be read from these figures. Third, the choice of priors for treatment effects can be criticised as being overly subjective. However, using a ‘family of priors’ approach with different beliefs of treatment effect, the impact of prior choices is easily interpreted through comparisons.^4^ Fourth, we present an effect modelling approach to HTE that has several limitations, including the risk of false negative and false positive results.^11^ However, the HTE analyses presented have *high credibility* according to the ICEMAN checklist. Fifth, we did not investigate HTE in renal replacement therapy groups, which could impact the relationship between protein, AKI, and outcomes.^23^^,^^25^^,^^28^ We decided that a limited number of participants receiving renal replacement therapy and inherent variation on clinical decisions to start renal replacement therapy would limit the inference from such analyses. Finally, although the Bayesian analyses may give rise to a more nuanced interpretation of the EFFORT Protein trial, these results share the limitations of the primary pragmatic randomised controlled trial design where the intervention was not blinded, an unplanned primary outcome alteration attributed to COVID-19 occurred, within-group variation in nutrition intake was reported, and 28 patients were excluded post-randomisation.

### Conclusions

We found moderate to high probabilities of harm and low probabilities of benefit with high protein doses compared with usual protein in ICU patients for the primary and secondary outcomes. We found suggestions of *heterogeneity in treatment effects* with worse outcomes in participants randomised to high protein doses with renal dysfunction or greater illness severity at baseline.

## Authors’ contributions

Study conception: RWH, JP

Statistical analysis plan and protocol: RWH, ZP, JP, DKH, AGD

Involved in the EFFORT Protein trial: AGD, DEB, DKH

Analyses: RWH, AG

Writing of the first draft: RWH

Critical revision: all authors

Approval of final manuscript: all authors

## Declarations of interest

DEB has received speaker fees, conference attendance support, or advisory board fees from Baxter, Cardinal Health, and Avanos. ZP has received honoraria for consultancy from GlaxoSmithKline, Lyric Pharmaceuticals, Faraday Pharmaceuticals, and Fresenius-Kabi; educational support from Baxter and Nestle Health Science; and speaker fees from Orion, Baxter, Sedana, Fresenius-Kabi, and Nestle. JP has recent consultancy agreements with Jaffron Biomedical, Mission Therapeutics, Paion Ltd, Nephrolyx GmbH, Medibeacon Inc., Baxter Inc., and Nikkiso Europe GmbH; has received speaker's fees and hospitality from Baxter Inc., BBraun, Nikkiso Europe, and Fresenius Medical Care; and has received research support from Astute Medical/Biomerieux and Jaffron Biomedical. AG has received research funding from Sygeforsikringen ‘danmark’ and the Novo Nordisk Foundation. The remaining authors have no competing interest to declare.
