# Supplementary material for: The effect of high protein dosing in critically ill patients: an exploratory, secondary Bayesian analyses of the EFFORT Protein trial
Source: Br J Anaesth. 2024 Oct 24;133(6):1192–200. doi: 10.1016/j.bja.2024.08.033 (PMC11589476; doi:10.1016/j.bja.2024.08.033)
Supplement: Multimedia component 1 [file mmc1.docx]

**The effect of high protein dosing in critically ill patients - Bayesian analyses of the EFFORT Protein trial**

**Electronic Supplementary Material**

Ryan W. Haines PhD, Anders Granholm MD, Zudin Puthucheary PhD, Andrew Day MD, Danielle E. Bear PhD, John R. Prowle MD, Daren K. Heyland MD

**Enrolment criteria in EFFORT-Protein (The effect of higher protein dosing in critically ill patients with high nutritional risk)**

Additional details available in the published trial.[1]

*Inclusion criteria*

Adult patients (≥18 years) within 96 h of intensive care (ICU) admission who were expected to remain mechanically ventilated for at least 48 h from screening with one or more of the following nutritional risk factors: low (≤25 kg/m2) or high (≥35 kg/m2) body mass index (BMI); moderate to severe malnutrition, as defined by local assessments; frailty, as defined by a Clinical Frailty Scale (version 2) of 5 or more from proxy; sarcopenia, as defined by a SARC-F (sarcopenia five questionnaire) score of 4 or more from proxy; and from point of screening, projected duration of mechanical ventilation of more than 4 days.

*Exclusion criteria*

Patients who had received more than 96 continuous hours of mechanical ventilation before screening, those expected to die or undergo withdrawal of life-sustaining treatments within 7 days from screening, pregnant women, patients for whom the responsible clinician felt that the patient either needed low or high protein (no clinical equipoise), and patients who required parenteral nutrition only in which the site did not have products to reach the high protein dose targets.

**Detailed outcome definitions**

*Primary outcome:*

60-day all-cause mortality: death from any cause within 60 days after randomisation.

*Secondary outcome:*

Time-to-discharge alive from hospital up to 60 days post randomisation.

The original primary outcome for EFFORT-Protein was 60-day mortality and the secondary outcome was time-to-discharge-alive from hospital. However, due to the COVID-19 pandemic, enrolment decreased substantially and achieving the original sample size was not feasible. Accordingly, with an expectation that the COVID-19 pandemic would persist and the trial would enrol at least 1200 patients, the new primary outcome was changed to time-to-discharge alive from hospital.[1]

**Methodological details**

All analyses were based on the internal protocol prepared after the results of the EFFORT-Protein trial were available (see Appendix). We altered the modelling approach in HTE analyses as outlined in detail in the relevant section below but in brief; did not add extra random effects in hierarchical models when modelling HTE for only two groups (SOFA and AKI analyses) due to limited benefit over use of a sceptical prior on the interaction term, log2 transformed baseline creatinine values to aid modelling properties, and included AKI staging in HTE analyses due to a recent re-analysis of EFFORT-Protein.

*Model specifications*

All models were developed using an approach adopted from analyses of the CLASSIC trial by Sivapalan et al [2, 3]. Like the IV fluid intervention (standard vs. restrictive) in CLASSIC, nutritional supplementation is very commonly administered when caring for critically ill patients and therefore small differences in outcomes with a ubiquitous therapy are potentially important.

All models were analysed using non-centred parameterisations of the intercept. Of the 85 sites, 52 recruited <12 patients. To avoid issues with nuisance parameters in Bayesian models we merged with sites recruiting <12 patients to sites in the same city or if not possible, region.[4]

60-day all-cause mortality was analysed using hierarchical Bayesian logistic regression models (*family = Bernoulli(link = “logit”)*) in the brms R package[5] adjusted for the stratification variable (trial site) as a random effect. Below the brms formula syntax is specified:

*bf(outcome ~ 1 + treatment + (1|site), centre = FALSE)*

Here, the *bf* function sets up the brms model formula using multiple arguments, outcome corresponds to 60-day mortality; *1* signifies the intercept, representing the baseline risk for a patient with all additional variables set to their reference values; *treatment* represent the intervention effect with high protein versus usual protein therapy, where the latter serves as the reference; *site* denotes the participating sites, treated as random effects; *center=FALSE* term signifies the usage of regular (non-centred) intercepts.

Time-to-discharge alive was analysed using Bayesian regression models (family= "cox") in the brms package adjusted for the stratification variables with the following formula syntax:

*bf(Time-to-discharge-alive | censored) ~ 1 + treatment + site, centre = FALSE)*

Here, time-to-discharge-alive denotes the absolute number of days alive until hospital discharge, up to 60 days post randomisation. Participants that died are censored at the maximum days (60) as this approximates the Fine and Gray substitution hazard approach. In the time-to-discharge-alive model site was a fixed effect with reference set to the most common category, which was the Mexico Hospital Civil Fray Antonio Alcalde. We avoided random effects in the Cox model to allow easier sampling and interpretation of the posterior.

*Heterogeneity of treatment effects (HTE) on 60-day mortality*

We examined HTE on 60-day mortality based on the following baseline variables: 1) presence of acute kidney injury (AKI), 2) highest creatinine value, 3) overall severity of illness using the Sequential Organ Failure Assessment (SOFA) high (≥9) versus low (<9), 4) SOFA score on the continuous scale (0-24), 5) BMI high (>30) versus low (≤30) and 6) BMI on the continuous scale).

All HTE analyses were analysed using adjusted hierarchical Bayesian logistic regression models with random effects for sites. We used an interaction term included for each baseline variable adjusted for stratification variable (site) with the following brms formula syntax:

*bf(mortality ~ 1 + treatment + variable + treatment:variable + (1|new_site), centre = FALSE)*

Here, *treatment:variable* is the interaction term for the baseline variable at randomisation and treatment effect of high protein. Creatinine was mean centred, log2 transformed, and modelled as linear. All other parameters are as defined above.

**Priors**

The following is a summary of the priors used in the conducted analyses in line with the specifications in the protocol. For the primary analyses we used weakly informative or sceptical priors centred on no difference and including all plausible effect sizes. Sensitivity analyses were conducted with optimistic and pessimistic priors of moderate belief, whilst keeping the same weakly informative priors for all other parameters.

*Primary outcome*

The family of priors for 60-day all-cause mortality are presented in figure S1.[6] We used a *normal(mean = -0.40, standard deviation [SD] = 1.5)* prior for the intercept corresponding to a probability baseline risk centred on 40% with 95% probability mass between 3% and 93% for the usual protein group (control group). For the group level effect of trial site we used a *normal(0, omega)* prior. Here, omega is the shrinkage factor estimated from the data by using a half-normal prior for omega with an SD of 1. [3, 7]

*Secondary outcome*

The family of priors for time-to-discharge alive from hospital are presented in figure S2. The revised EFFORT-Protein primary outcome powered for a 20% increase in hazard of time-to-discharge alive from hospital.[1] Therefore, optimistic prior was centred on 20% increase (-15% to 55%) and pessimistic prior a 20% (-55% to 15% )decrease hazard of alive hospital discharge. Other parameters were set using default brms priors for intercept *student(degrees of freedom: 3, mu: 3.9, sigma: 2.5)*, baseline hazard *Dirichlet(1)*, and correlated random effects *Cholesky LKJ*(1) [5]

*HTE analyses*

We used the same priors as the primary outcome analysis (sceptical prior for treatment effect). We used priors for AKI and SOFA groups interaction effect that were sceptical of any group effects: *normal(mean = 0, SD = 0.1).* We used less sceptical priors for effect of AKI and SOFA: *normal(mean = 0, SD = 0.5).*

For the creatinine analysis, creatinine values were mean centred and transformed to log base 2. Priors were defined as *normal(0, x)* with *x* being 1/SD of the log2-transformed creatinine. Therefore, we expect a priori that changes on the log odds scale are +/- 1.96 across 95% of creatinine change. The same prior was used for the interaction effect. For the SOFA score analyses priors were defined as *normal(0, x)* with x being 1/SD and the same prior used for the interaction effect. For the BMI analyses priors were defined as *normal(0, x)* with x being 1/SD and the same prior used for the interaction effect.

**Model diagnostics**

We used Stan’s default dynamic Hamiltonian Monte Carlo sampler with four chains with 5,000 warm-up samples per chain and 20,000 post-warm-up samples in total and at least 10,000 bulk/tail effective sample sizes for the treatment effect parameter(s) and 1000 bulk/tail effective sample sizes for all other parameters. We visually assessed density and trace plots for chain convergence and required Rhat statistics ≤ 1.01 for all parameters.[8, 9]

Figure S1 Prior distributions for 60-day mortality models.


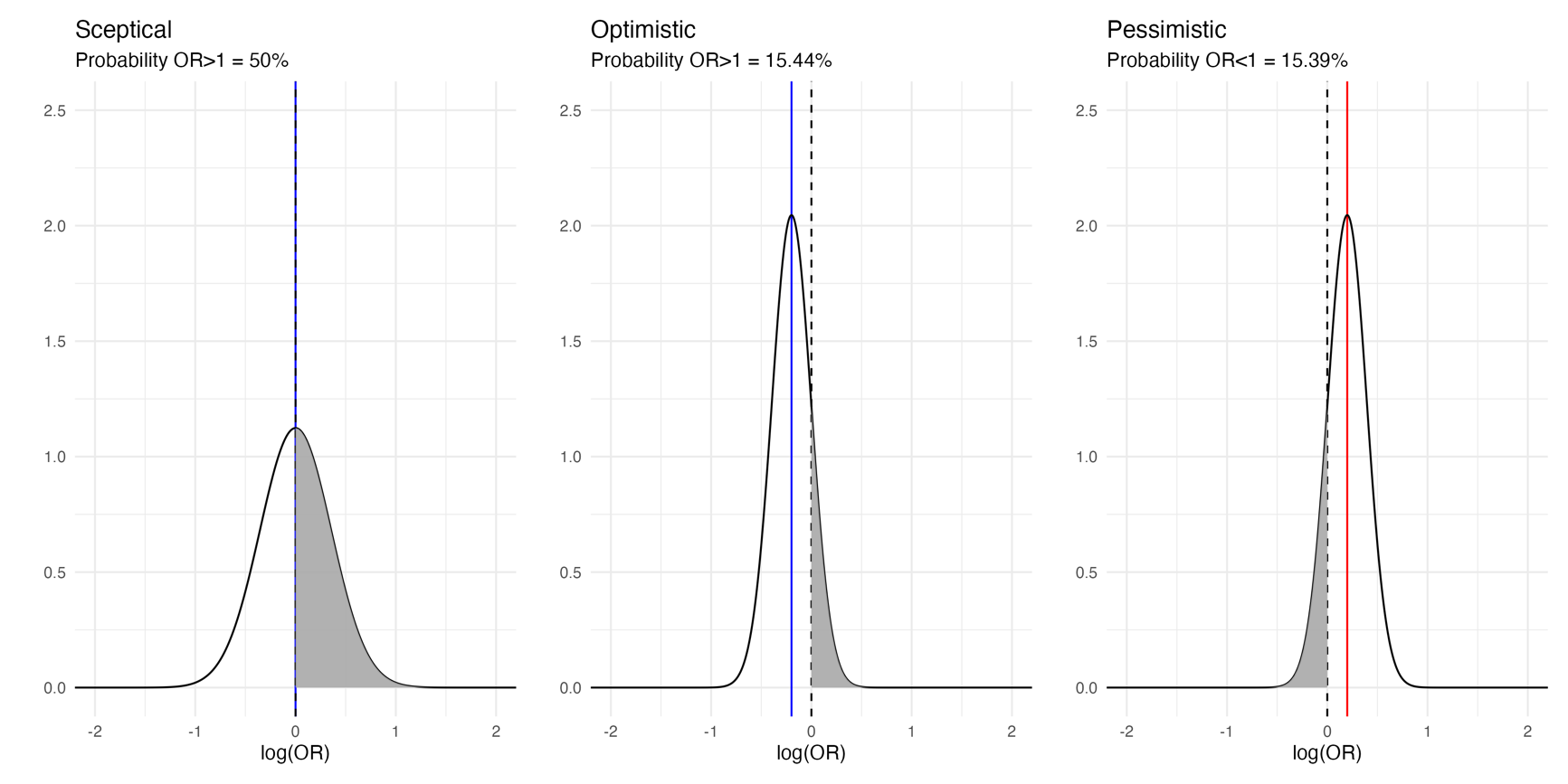


Left panel: Sceptical prior - N(0, 0.355). Middle panel: Optimistic prior - N(−0.198, 0.195). Right panel: Pessimistic prior - N(0.198, 0.195). All priors of moderate strength belief, for example; for the sceptical prior: “I have no reason to believe the intervention is good or bad, but I am mostly sure I can rule out large effect sizes.” Priors summarised as N(μ, σ), which indicates a normal distribution with mean = μ and SD = σ. The prior is for the log(OR) of the intervention.[6]

Figure S2 Prior distributions for time-to-discharge-alive from hospital models.


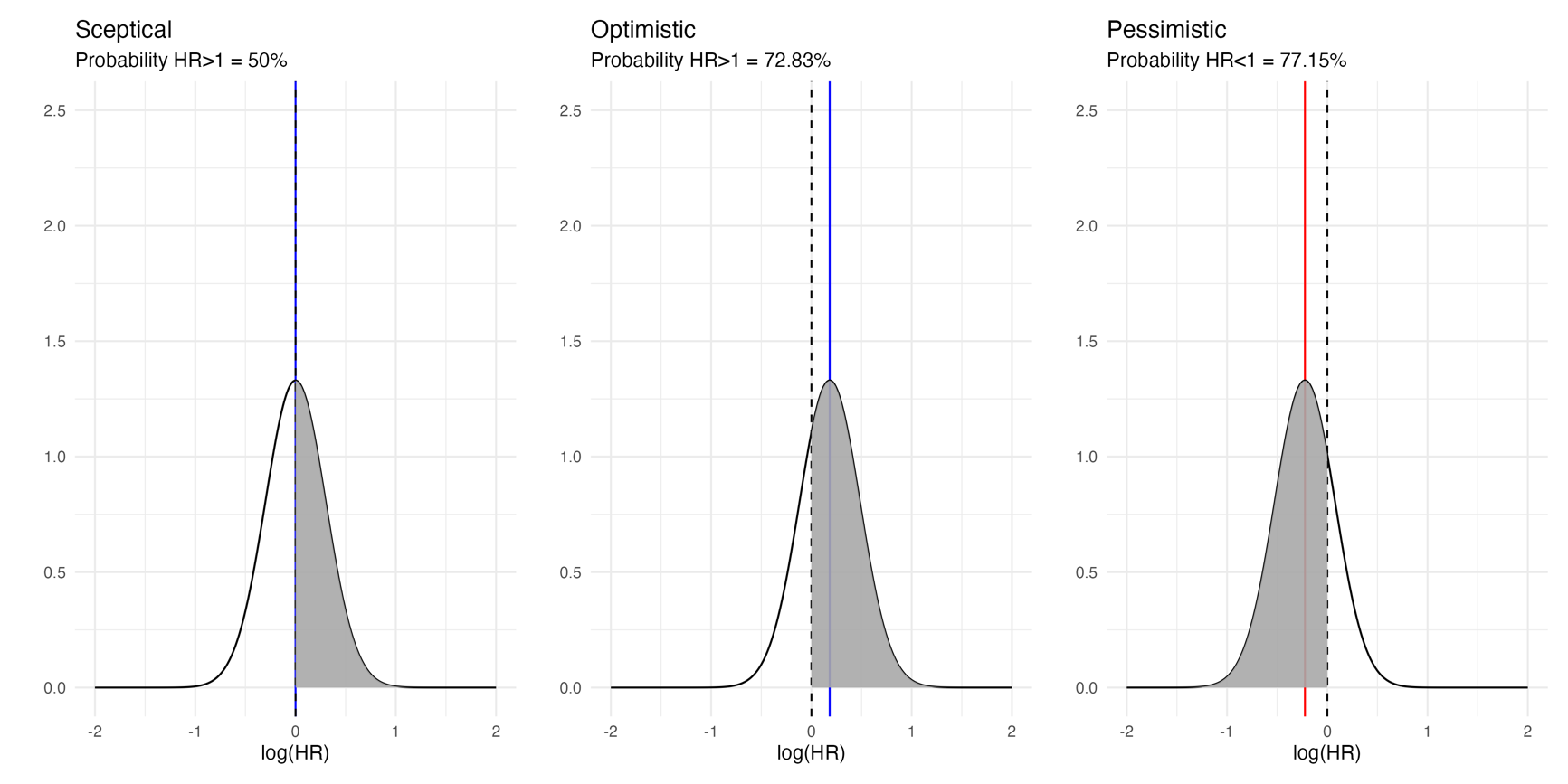


Left panel: Sceptical prior - N(0, 0.3). Middle panel: Optimistic prior - N(0.182, 0.3). Right panel: Pessimistic prior - N(-0.223, 0.3). All priors of moderate strength belief, for example; for the sceptical prior: “I have no reason to believe the intervention is good or bad, but I am mostly sure I can rule out large effect sizes.” Priors summarised as N(μ, σ), which indicates a normal distribution with mean = μ and SD = σ. The prior is for the log(HR) of the intervention.[6]

Table S1 Baseline characteristics and descriptive outcome data stratified by treatment.

|  |  | **Usual protein** | **High protein** | **All patients** |
| --- | --- | --- | --- | --- |
|  | N | (N=653) | (N=644) | (N=1297) |
| Age, years | 1297 | 45/**59**/70 | 47/**59**/69 | 46/**59**/69 |
| Female | 1296 | 41% 267/652 | 39% 249/644 | 40% 516/1296 |
| **Admission category:** |  |  |  |  |
| Medical | 1297 | 83% 539/653 | 85% 548/644 | 84% 1087/1297 |
| Surgical elective |  | 3% 19/653 | 4% 24/644 | 3% 43/1297 |
| Surgical emergency |  | 15% 95/653 | 11% 72/644 | 13% 167/1297 |
| COVID-19 positive on admission | 1297 | 7% 48/653 | 6% 37/644 | 7% 85/1297 |
| BMI | 1297 | 23/**26**/33 | 22/**26**/32 | 23/**26**/32 |
| Charlson Comorbidity Index | 1297 | 0/**0**/1 | 0/**0**/2 | 0/**0**/1 |
| Baseline SOFA score | 1297 | 6/**9**/11 | 6/**9**/11 | 6/**9**/11 |
| APACHE II score | 1226 | 15/**21**/26 | 16/**21**/27 | 15/**21**/26 |
| mNUTRIC score | 1226 | 3/**5**/6 | 3/**5**/6 | 3/**5**/6 |
| Frailty | 1192 | 2/**3**/4 | 2/**3**/5 | 2/**3**/4 |
| SARC-F score | 1164 | 0/**1**/4 | 0/**1**/5 | 0/**1**/5 |
| Renal replacement therapy on randomisation day | 1297 | 15% 98/653 | 19% 121/644 | 17% 219/1297 |
| Serum creatinine, μmol/L | 1207 | 65/**89**/133 | 64/**97**/141 | 65/**92**/140 |
| **Acute kidney injury at time of randomisation*:** | 1277 |  |  |  |
| Yes |  | 23% 147/653 | 25% 162/644 | 24% 309/1297 |
| Stage 1 |  | 40% 59/147 | 36% 58/162 | 38% 117/309 |
| Stage 2 |  | 21% 31/147 | 27% 43/162 | 24% 74/309 |
| Stage 3 |  | 39% 57/147 | 38% 61/162 | 38% 118/309 |
| Moderate or severe chronic renal disease^$^ | 1297 | 8% 51/653 | 10% 63/644 | 9% 114/1297 |
| ICU length of stay | 1291 | 5/**9**/19 | 6/**10**/18 | 5/**10**/19 |
| Hospital length of stay | 1289 | 10/**19**/38 | 9/**19**/39 | 10/**19**/38 |
| 30-day mortality | 1297 | 26% 171/653 | 28% 181/644 | 27% 352/1297 |
| 60-day mortality | 1297 | 32% 207/653 | 34% 221/644 | 33% 428/1297 |

*a*/***b***/*c* represent the lower quartile *a*, the median *b*, and the upper quartile *c* for continuous variables. Categorical variables are described by counts and percentages. *N* is the number of non-missing values. APACHE=Acute Physiology and Chronic Health Evaluation. ICU=intensive care unit. SOFA=Sequential Organ Failure Assessment. mNUTRIC=modified Nutrition Risk Assessment in Critical Illness Score. SARC-F= five item sarcopenia questionnaire score.

*Acute kidney injury refers to participants who met the criteria of KDIGO: stage 1 is at least 26.52 μmol/L increase in serum creatinine from baseline within 48 h or 1.5–1.9 times baseline within 7 days; stage 2 is 2.0–2.9 times baseline within 7 days; stage 3 is three times or more baseline within 7 days or increase to at least 353.6 μmol/L with an acute increase of more than 44.2 μmol/L.

^$^Defined in comorbidities as moderaterenal disease:creatinine clearance 51–85 mL/min; and severe renal disease: creatinine clearance less than 50 mL/min and not on dialysis.

Figure S3 Posterior probability distribution of relative risk for 60-day all-cause mortality in the sensitivity analysis using optimistic priors.


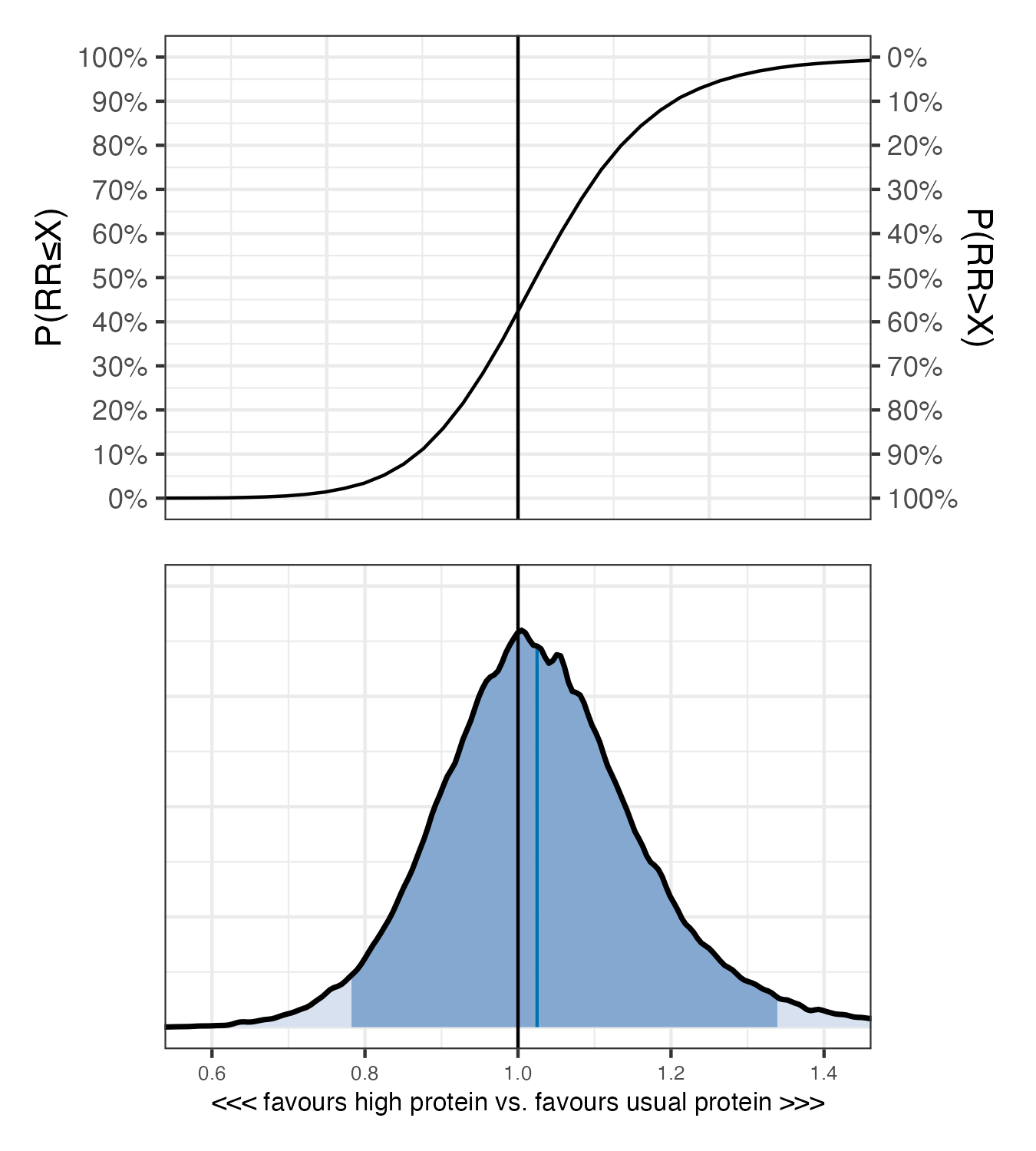


Posterior probability distribution of the conditional risk ratios (RR) for 60-day all-cause mortality in the primary analysis using optimistic priors, adjusted for trial site (stratification variable). The black vertical line represents exactly no difference. The plot displays the relative difference, where an RR < 1.00 favours the higher protein dose group while an RR > 1.00 favours the usual protein group. Top panel: cumulative posterior distribution of effect sizes. Bottom panel: corresponding posterior density plot with median (blue vertical line) and percentile-based 95% credible interval (blue area). X denotes various treatment effect sizes on the horizontal axis with the corresponding probabilities of RR ≤ X values on the left Y-axis and the RR > X on the right Y-axis.

Figure S4 Posterior probability distribution for the risk differences for 60-day all-cause mortality in the sensitivity analysis using optimistic priors.


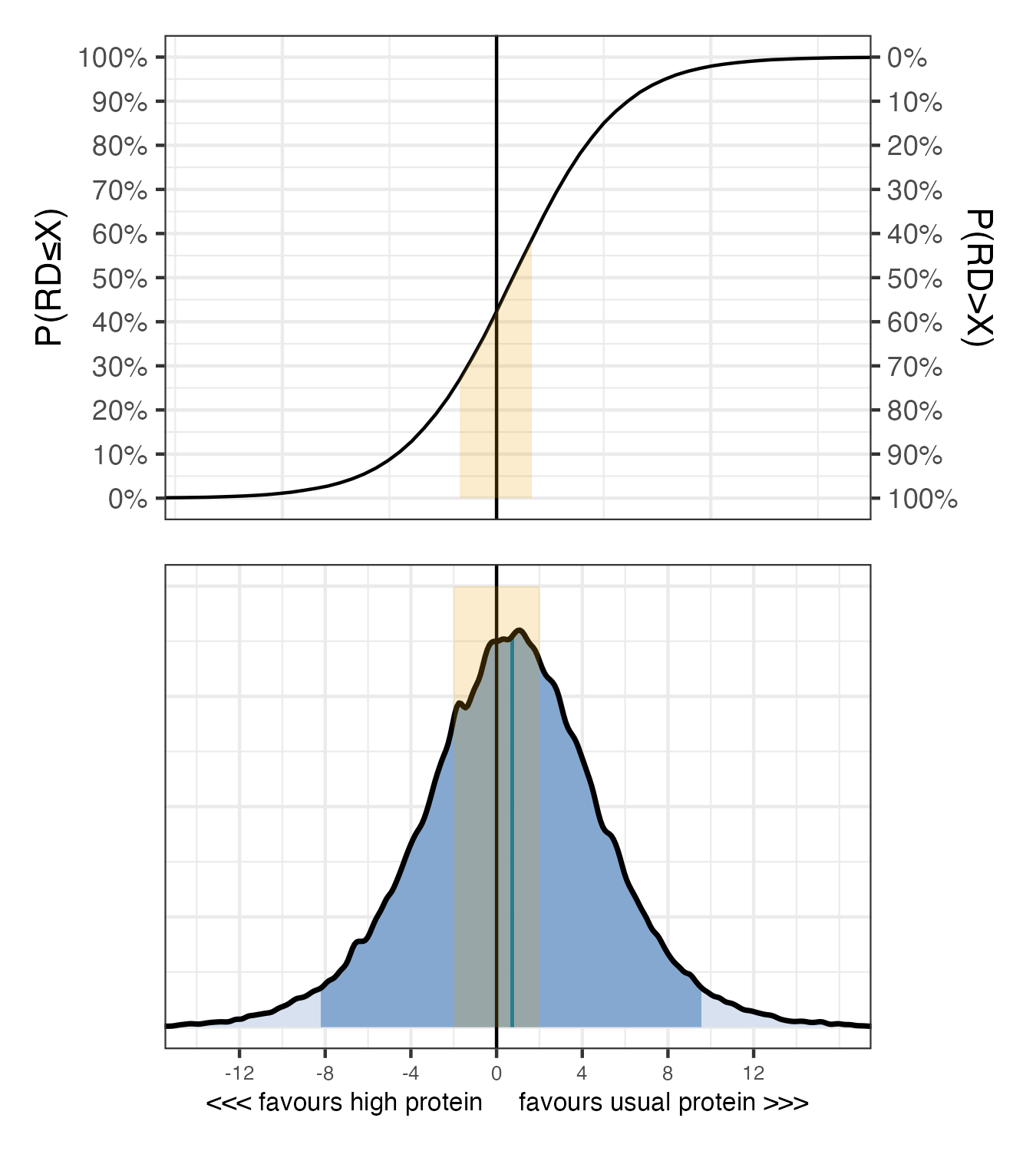


Posterior probability distributions for 60-day mortality using optimistic priors adjusted for trial site (random effect). The black vertical lines represent no difference. Top panel: cumulative posterior distribution of effect sizes. Bottom panel: corresponding posterior density plot with median (blue vertical line) and percentile-based 95% credible interval (CrI, blue area). Yellow area demarks effect sizes smaller than the pre-defined minimally clinically important effect. X denotes various treatment effect sizes on the horizontal axis with the corresponding probabilities of RD ≤ X values on the left Y-axis and the RD > X on the right Y-axis.

Figure S5 Posterior probability distribution of relative risk for 60-day all-cause mortality in the sensitivity analyses using pessimistic priors.


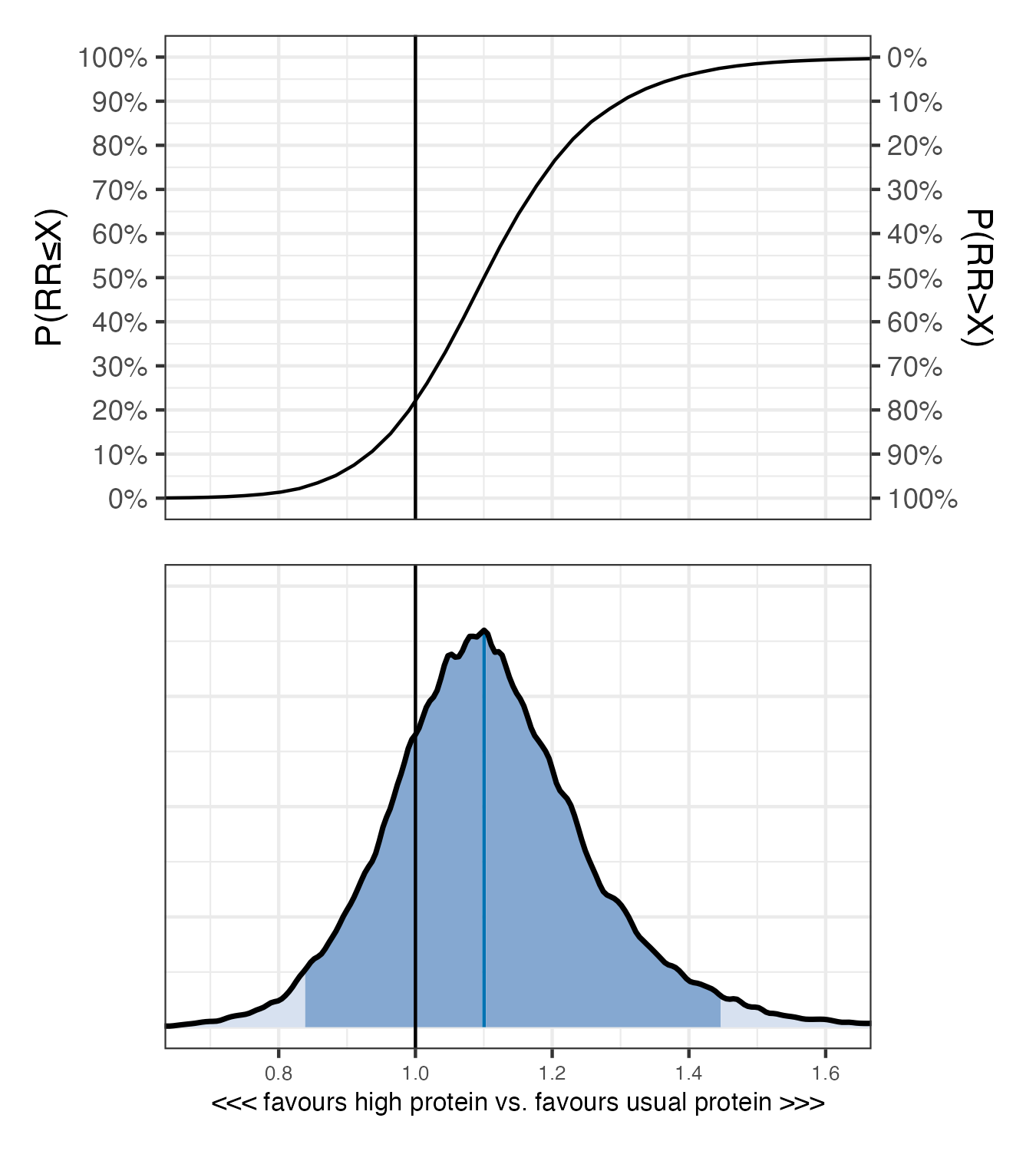


Posterior probability distribution of the conditional risk ratios (RR) for 60-day all-cause mortality in the primary analysis using pessimistic priors, adjusted for trial site (stratification variable). The black vertical line represents exactly no difference. The plot displays the relative difference, where an RR < 1.00 favours the higher protein dose group while an RR > 1.00 favours the usual protein group. Top panel: cumulative posterior distribution of effect sizes. Bottom panel: corresponding posterior density plot with median (blue vertical line) and percentile-based 95% credible interval (blue area). X denotes various treatment effect sizes on the horizontal axis with the corresponding probabilities of RR ≤ X values on the left Y-axis and the RR > X on the right Y-axis.

Figure S6 Posterior probability distribution for the risk differences for 60-day all-cause mortality in the sensitivity analysis using pessimistic priors.


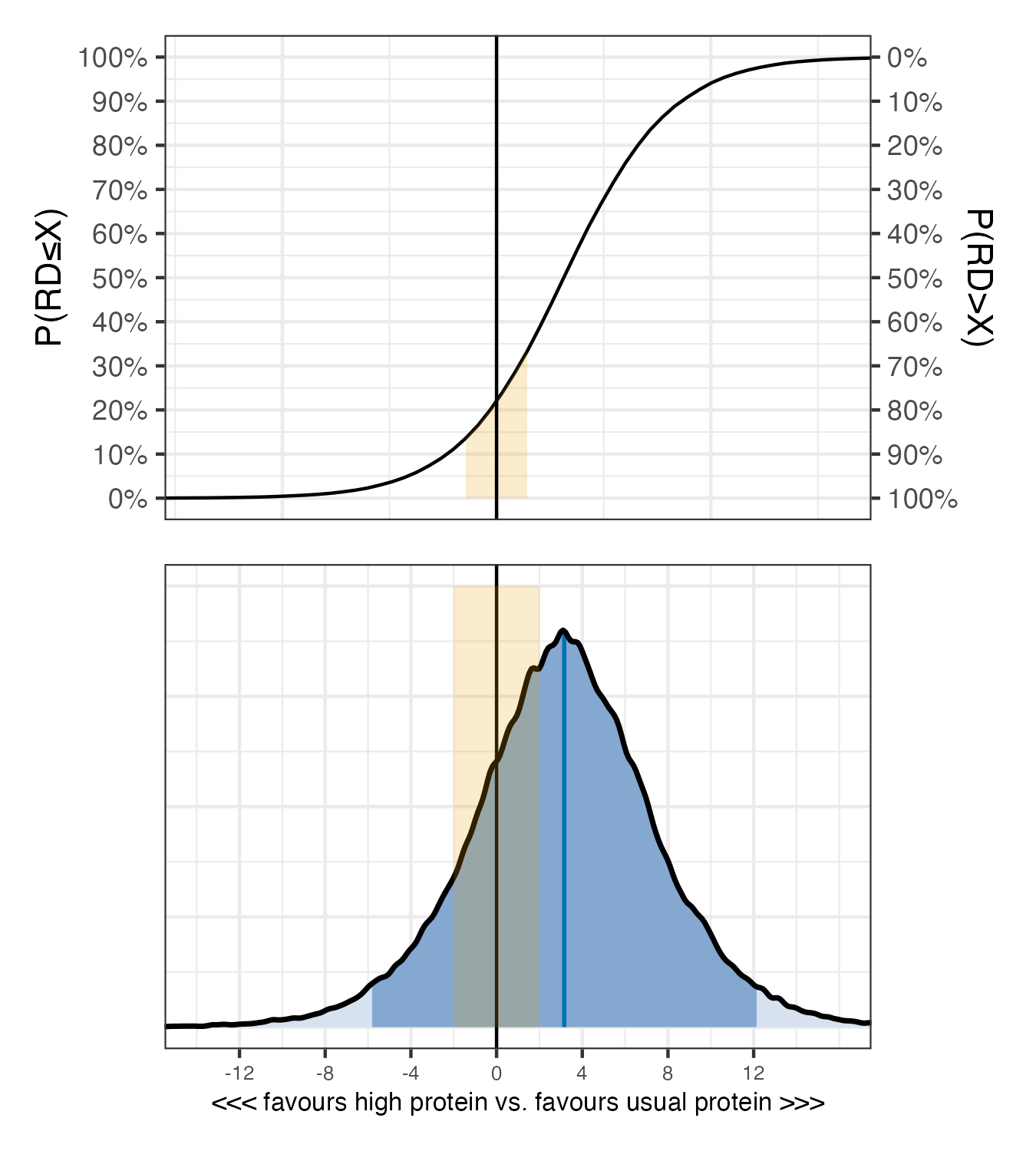


Posterior probability distributions for 60-day mortality using pessimistic priors adjusted for trial site (random effect). The black vertical lines represent no difference. Top panel: cumulative posterior distribution of effect sizes. Bottom panels: corresponding posterior density plot with median (blue vertical line) and percentile-based 95% credible interval (CrI, blue area). Yellow area demarks effect sizes smaller than the pre-defined minimally clinically important effect. X denotes various treatment effect sizes on the horizontal axis with the corresponding probabilities of RD ≤ X values on the left Y-axis and the RD > X on the right Y-axis.

Table S3 Results of Bayesian analysis of EFFORT-Protein trial – time-to-discharge-alive from hospital.

|  | Hazard ratio  (95% CrI) | Percentage difference in hazard ratio (95% CrI) | Probability of harm (hazard ratio <1) | Probability of benefit (hazard ratio >1) |
| --- | --- | --- | --- | --- |
| Secondary analysis using a weakly informative sceptical prior | 0.91  (0.80 to 1.04) | -8.8%  (-19.9 to 3.8) | 92% | 8% |
| Secondary analysis using an optimistic prior | 0.92  (0.81 to 1.05) | -7.9%  (-19.1 to 5.0) | 89% | 11% |
| Secondary analysis using a pessimistic prior | 0.90  (0.79 to 1.03) | -9.7%  (-20.6 to 2.9) | 94% | 6% |

Priors were set following a statistical analysis plan which used the suggested principles outlined by Harrel et al, using sceptical, optimistic, and pessimistic priors of moderate strength at N(1, 0.3), N(0.8, 0.3), and N(1.2, 0.3) respectively. Priors are shown in figure S2. Models adjusted for trial site (fixed effect). All analyses were conducted in the modified intention-to-treat population after exclusion of four participants due to missing primary outcome data (*n* = 1297). The effect estimates are reported as hazard ratios (HR) with median posterior values as point estimates and percentile-based 95% credible intervals (CrIs).

Abbreviations: Crl, credible interval; HR, hazard ratio.

Figure S7 Posterior probability distribution for time-to-discharge-alive from hospital.


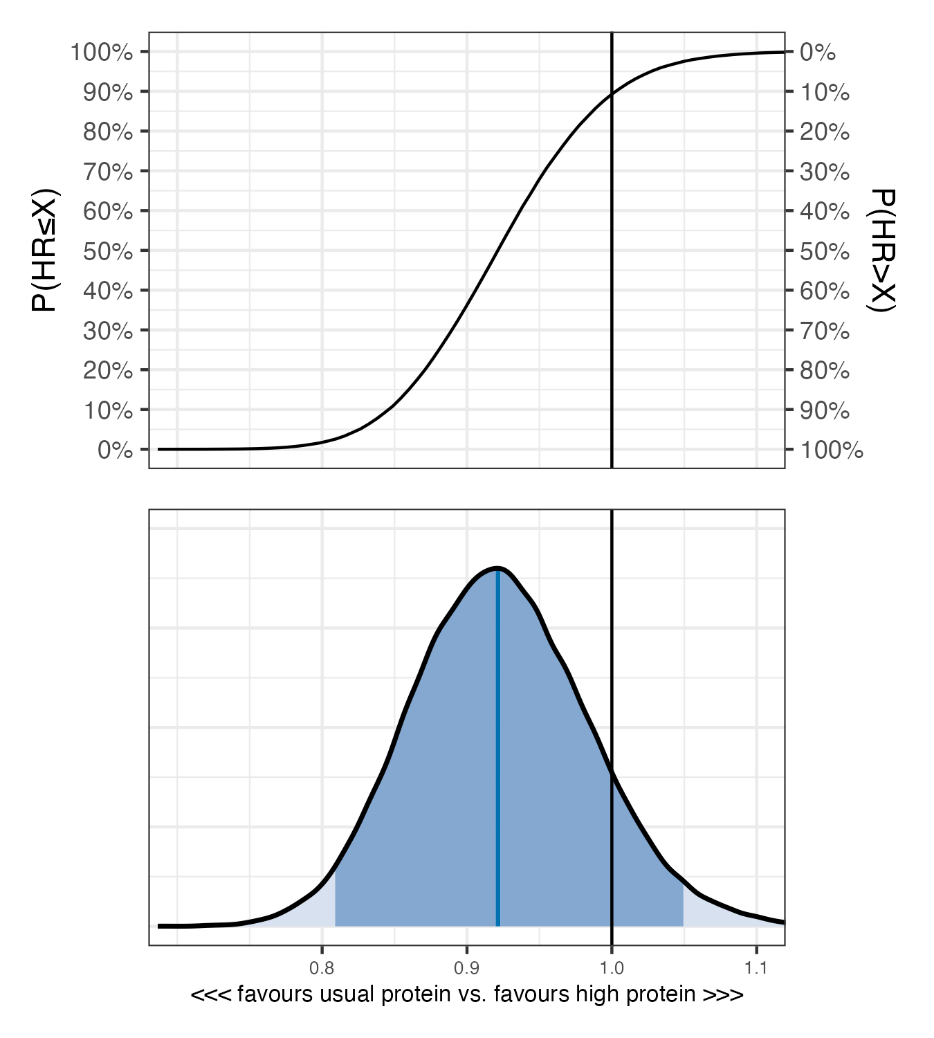


Models used weakly informative priors adjusted for trial site (fixed effect). The black vertical lines represent no difference. Top panel: cumulative posterior distribution of hazard ratios. Bottom panel: corresponding posterior density plot with median (blue vertical line) and percentile-based 95% credible interval (CrI, blue area). Optimistic prior (N(0.8, 0.3)), hazard ratio with a median of 0.92 (0.81 to 1.05). X denotes various treatment effect sizes on the horizontal axis with the corresponding probabilities of HR ≤ X values on the left Y-axis and HR > X on the right Y-axis.

Figure S8 Posterior probability distribution for time-to-discharge-alive from hospital.


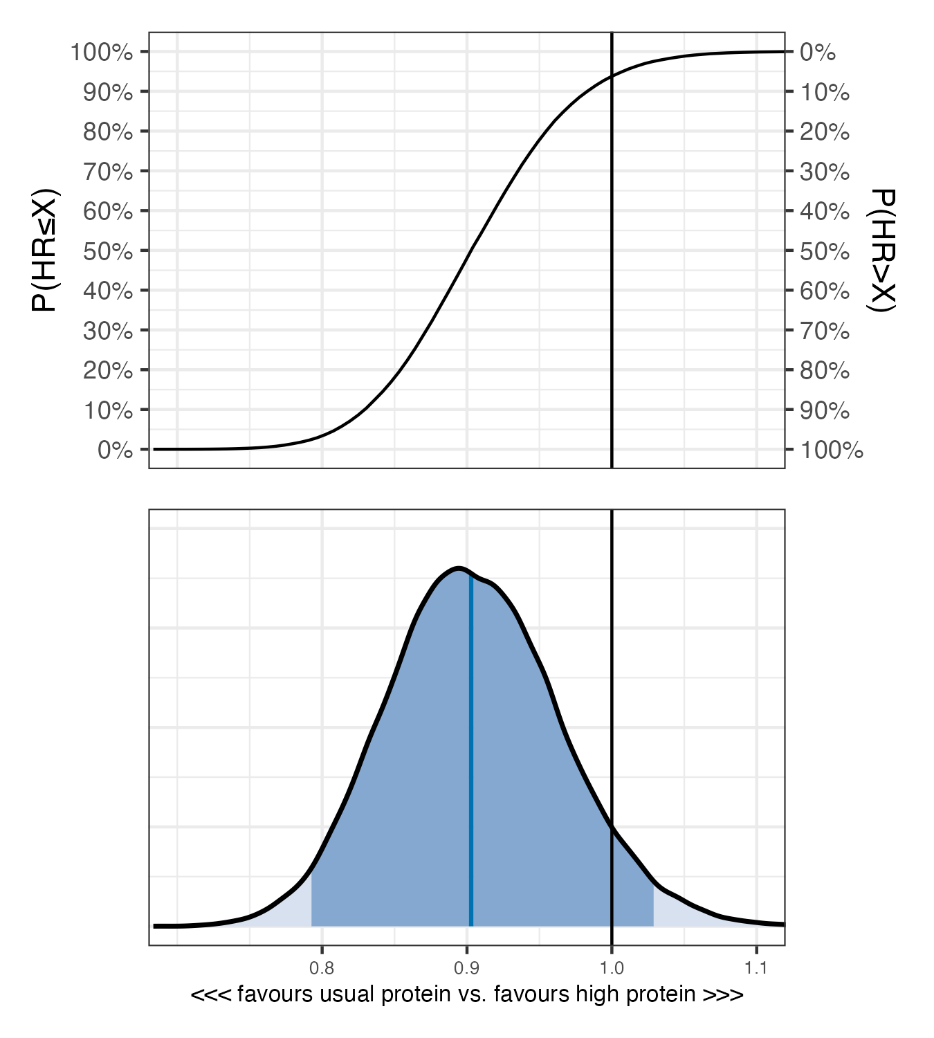


Models used weakly informative priors adjusted for trial site (fixed effect). The black vertical lines represent no difference. Top panel: cumulative posterior distribution of hazard ratios. Bottom panel: corresponding posterior density plot with median (blue vertical line) and percentile-based 95% credible interval (CrI, blue areas). Pessimistic prior (N(1.2, 0.3)), hazard ratio with a median of 0.90 (0.79 to 1.03). X denotes various treatment effect sizes on the horizontal axis with the corresponding probabilities of HR ≤ X values on the left Y-axis and HR > X on the right Y-axis.

Heterogeneity of treatment effects (HTE)

Table S4 Baseline characteristics and descriptive outcome data in the acute kidney injury (AKI) subgroups.

|  | **No Acute Kidney Injury** | | | **Acute Kidney Injury** | | |
| --- | --- | --- | --- | --- | --- | --- |
|  |  | Usual protein | High protein |  | Usual protein | High protein |
| **Characteristics** | N | (N=506) | (N=482) | N | (N=147) | (N=162) |
| Age, years | 988 | 43/**58**/69 | 45/**58**/68 | 309 | 50/**62**/73 | 54/**62/**69 |
| Female | 988 | 41% 208/506 | 39% 189/482 | 308 | 40% 59/146 | 37% 60/162 |
| Admission category: |  |  |  |  |  |  |
| Medical | 988 | 80% 406/506 | 84% 404/482 | 309 | 90% 133/147 | 89% 144/162 |
| Surgical elective |  | 3% 13/506 | 4% 19/482 |  | 4% 6/147 | 3% 5/162 |
| Surgical emergency |  | 17% 87/506 | 12% 59/482 |  | 5% 8/147 | 8% 13/162 |
| COVID-19 positive on admission | 988 | 8% 40/506 | 6% 29/482 | 309 | 5% 8/147 | 5% 8/162 |
| BMI | 988 | 23.0/**26.4**/32.3 | 22.3/**25.6**/31.2 | 309 | 22.1/**25.6**/34.0 | 22.2/**26.8**/33.2 |
| Charlson Comorbidity Index | 988 | 0/**0**/1 | 0/**0**/1 | 309 | 0/**1**/2 | 0/**1**/2 |
| Baseline SOFA score | 988 | 6/**8**/11 | 6/**8**/11 | 309 | 7/**10**/13 | 8/**10**/12 |
| APACHE II score | 931 | 15/**20**/25 | 15/**20**/26 | 295 | 18/**25**/29 | 19/**23**/29 |
| mNUTRIC score | 931 | 3/**4**/6 | 3/**4**/6 | 295 | 4/**6**/7 | 4/**5**/7 |
| Frailty | 910 | 2/**3**/4 | 2/**3**/4 | 282 | 3/**4**/5 | 3/**4**/5 |
| SARC-F score | 884 | 0/**1**/4 | 0/**1**/4 | 280 | 0/**2**/6 | 0/**3**/6 |
| Moderate or severe chronic renal disease^$^ | 988 | 5% 24/506 | 6% 28/482 | 309 | 18% 27/147 | 22% 35/162 |
| ICU length of stay | 983 | 5.1/ **9.9**/20.0 | 5.8/**10.1**/18.2 | 308 | 5.1/**8.5**/14.22 | 5.0/**9.1**/18.5 |
| Hospital length of stay | 981 | 10.1/**19.1**/37.9 | 10.1/**20.9**/40.3 | 308 | 8.6/**18.8**/36.0 | 7.6/**17.5**/37.1 |
| 30-day mortality | 988 | 25% 125/506 | 23% 112/482 | 309 | 31% 46/147 | 43% 69/162 |
| 60-day mortality | 988 | 31% 155/506 | 29% 139/482 | 309 | 35% 52/147 | 51% 82/162 |

*a* ***b*** *c* represent the lower quartile *a*, the median *b*, and the upper quartile *c* for continuous variables. Categorical variables are described by counts and percentages. *N* is the number of non-missing values. APACHE=Acute Physiology and Chronic Health Evaluation. ICU=intensive care unit. SOFA=Sequential Organ Failure Assessment. SARC-F= five item sarcopenia questionnaire score. mNUTRIC=modified Nutrition Risk Assessment in Critical Illness Score. Acute kidney injury refers to participants who met the criteria of KDIGO: stage 1 is at least 26.52 μmol/L increase in serum creatinine from baseline within 48 h or 1.5–1.9 times baseline within 7 days; stage 2 is 2.0–2.9 times baseline within 7 days; stage 3 is three times or more baseline within 7 days or increase to at least 353.6 μmol/L with an acute increase of more than 44.2 μmol/L.

^$^Defined in comorbidities as moderate [renal disease](https://www.sciencedirect.com/topics/medicine-and-dentistry/nephropathy): [creatinine clearance](https://www.sciencedirect.com/topics/medicine-and-dentistry/creatinine-clearance) 51–85 mL/min; and severe renal disease: creatinine clearance less than 50 mL/min and not on dialysis.

Table S5 Baseline characteristics and descriptive outcome data in baseline serum creatinine quartiles.

| Creatinine ranges  (μmol/L) | Creatinine: 12-65 | | | Creatinine: 66-92 | | | | Creatinine: 93-139 | | | | Creatinine: 140-2400 | | | |
| --- | --- | --- | --- | --- | --- | --- | --- | --- | --- | --- | --- | --- | --- | --- | --- |
|  | **N** | **Usual protein** | **High protein** | | **N** | **Usual protein** | **High protein** | | **N** | **Usual protein** | **High protein** | | **N** | **Usual protein** | **High protein** |
| Characteristics |  | **(N=150)** | **(N=157)** | |  | **(N=168)** | **(N=131)** | |  | **(N=146)** | **(N=153)** | |  | **(N=142)** | **(N=160)** |
| Age, years | 307 | 43/**58**/68 | 46/**58**/68 | | 299 | 42/**58**/69 | 43/**56**/68 | | 299 | 42/**59**/71 | 50/**60**/70 | | 302 | 50/**61**/70 | 52/**61**/69 |
| Female | 307 | 51%  77/150 | 55%  86/157 | | 299 | 46%  77/168 | 34%  44/131 | | 298 | 28% 40/145 | 34%  52/153 | | 302 | 39% 56/142 | 33%  53/160 |
| Admission category: | |  |  | |  |  |  | |  |  |  | |  |  |  |
| Medical | 307 | 81%  121/150 | 83%  130/157 | | 299 | 82%  138/168 | 85%  111/131 | | 299 | 83%  121/146 | 86%  131/153 | | 302 | 87% 123/142 | 90% 144/160 |
| Surgical elective |  | 3%  4/150 | 8%  13/157 | |  | 4%  7/168 | 4%  5/131 | |  | 4%  6/146 | 3%  4/153 | |  | 1%  2/142 | 1%  2/160 |
| Surgical emergency |  | 17%  25/150 | 9%  14/157 | |  | 14%  23/168 | 11%  15/131 | |  | 13%  19/146 | 12%  18/153 | |  | 12%  17/142 | 9%  14/160 |
| COVID-19 positive on admission | 307 | 8%  12/150 | 7%  11/157 | | 299 | 11%  19/168 | 8%  10/131 | | 299 | 7%  10/146 | 7%  11/153 | | 302 | 3%  4/142 | 3%  4/160 |
| BMI | 307 | 22/**25**/32 | 20/**24**/31 | | 299 | 22/**27**/33 | 23/**26**/30 | | 299 | 23/**26**/32 | 23/**27**/34 | | 302 | 24/**27**/35 | 23/**27**/33 |
| Charlson Comorbidity Index | 307 | 0/**0**/1 | 0/**0**/1 | | 299 | 0/**1**/1 | 0/**0**/1 | | 299 | 0/**0**/1 | 0/**1**/2 | | 302 | 0/**1**/2 | 0/**1**/2 |
| Baseline SOFA score | 307 | 5/**7**/11 | 4/**7**/10 | | 299 | 6/**8**/10 | 5/**7**/10 | | 299 | 7/**9**/11 | 7/**9**/11 | | 302 | 9/**11**/14 | 8/**11**/13 |
| APACHE II score | 285 | 14/**19**/23 | 15/**19**/22 | | 283 | 14/**18**/24 | 13/**19**/24 | | 287 | 16/**21**/25 | 15/**21**/26 | | 295 | 21/**26**/30 | 21/**26**/30 |
| mNUTRIC score | 285 | 3/**4**/5 | 3/**4**/5 | | 283 | 3/**4**/5 | 3/**4**/5 | | 287 | 3/**5**/6 | 4/**5**/6 | | 295 | 5/**6**/7 | 5/**6**/7 |
| Frailty | 280 | 2/**3**/4 | 2/**3**/5 | | 277 | 2/**3**/4 | 2/**3**/4 | | 275 | 2/**3**/4 | 2/**3**/4 | | 275 | 3/**3**/5 | 2/**3**/4 |
| SARC-F score | 271 | 0/**1**/5 | 0/**2**/6 | | 274 | 0/**1**/4 | 0/**0**/3 | | 266 | 0/**0**/4 | 0/**1**/5 | | 268 | 0/**2**/5 | 0/**2**/4 |
| Renal replacement therapy on randomisation day | 307 | 5%  7/150 | 3%  4/157 | | 299 | 4%  6/168 | 6%  8/131 | | 299 | 13%  19/146 | 16%  25/153 | | 302 | 42% 59/142 | 46% 73/160 |
| Acute kidney injury at time of randomisation*: | 307 |  |  | | 299 |  |  | | 299 |  |  | | 302 |  |  |
| Yes |  | 12%  18/150 | 13%  21/154 | |  | 12%  20/168 | 16%  21/131 | |  | 27%  39/146 | 26%  39/153 | |  | 44%  63/142 | 43%  69/160 |
| Stage 1 |  | 28%  5/18 | 48%  10/21 | |  | 60%  12/20 | 57%  12/21 | |  | 49%  19/39 | 49%  19/39 | |  | 30%  19/63 | 22%  15/69 |
| Stage 2 |  | 33%  6/18 | 33%  7/21 | |  | 15%  3/20 | 33%  7/21 | |  | 28%  11/39 | 33%  13/39 | |  | 16%  10/63 | 17%  12/69 |
| Stage 3 |  | 39%  7/18 | 19%  4/21 | |  | 25%  5/20 | 10%  2/21 | |  | 23%  9/39 | 18%  7/39 | |  | 54%  34/63 | 61%  42/69 |
| Moderate or severe chronic renal disease^$^ | 307 | 0%  0/150 | 1%  1/157 | | 299 | 1%  1/168 | 2%  2/131 | | 299 | 6%  9/146 | 7%  10/153 | | 302 | 26%  37/142 | 28% 45/160 |
| Urea (mmol/L) | 298 | 3.5/**5.0**/6.7 | 3.3/**4.7**/6.3 | | 291 | 4.8/**6.5**/8.2 | 4.9/**6.4**/8.3 | | 292 | 6.0/**8.3**/11.1 | 6.4/**8.5**/10.9 | | 295 | 11.1/**15.4**/22.3 | 11.4/**16.7**/26.3 |
| Creatinine (μmol/) | 307 | 47/**55**/61 | 43/**53**/60 | | 299 | 71/**77**/84 | 70/**76**/84 | | 299 | 101/**110**/125 | 103/**110**/123 | | 302 | 168/**208**/ 293 | 162/**206**/ 330 |
| ICU length of stay | 306 | 5/**10**/21 | 7/**13**/24 | | 298 | 5/**9**/18 | 5/**9**/17 | | 296 | 5/**10**/19 | 6/**11**/22 | | 302 | 5/**8**/16 | 4/**8**/14 |
| Hospital length of stay | 306 | 10/**19**/36 | 12/**24**/47 | | 296 | 9/**18**/35 | 9/**18**/31 | | 296 | 11/**22**/36 | 10/**21**/41 | | 302 | 7/**16**/35 | 7/**15**/29 |
| 30-day mortality | 307 | 29%  43/150 | 22%  35/157 | | 299 | 25%  42/168 | 26%  34/131 | | 299 | 22%  32/146 | 25%  39/153 | | 302 | 29% 41/142 | 41%  65/160 |
| 60-day mortality | 307 | 32%  48/150 | 27%  43/157 | | 299 | 30%  51/168 | 32%  42/131 | | 299 | 28%  41/146 | 34%  52/153 | | 302 | 35% 50/142 | 46% 74/160 |

*a* ***b*** *c* represent the lower quartile *a*, the median *b*, and the upper quartile *c* for continuous variables. Categorical variables are described by counts and percentages. *N* is the number of non-missing values. Creatinine and urea are highest in 24 hours prior to randomisation. APACHE=Acute Physiology and Chronic Health Evaluation. ICU=intensive care unit. SOFA=Sequential Organ Failure Assessment. mNUTRIC=modified Nutrition Risk Assessment in Critical Illness Score. SARC-F= five item sarcopenia questionnaire score.

*Acute kidney injury refers to participants who met the criteria of KDIGO: stage 1 is at least 26.52 μmol/L increase in serum creatinine from baseline within 48 h or 1.5–1.9 times baseline within 7 days; stage 2 is 2.0–2.9 times baseline within 7 days; stage 3 is three times or more baseline within 7 days or increase to at least 353.6 μmol/L with an acute increase of more than 44.2 μmol/L.

^$^Defined in comorbidities as moderate [renal disease](https://www.sciencedirect.com/topics/medicine-and-dentistry/nephropathy): [creatinine clearance](https://www.sciencedirect.com/topics/medicine-and-dentistry/creatinine-clearance) 51–85 mL/min; and severe renal disease: creatinine clearance less than 50 mL/min and not on dialysis.

Table S6 Baseline characteristics and descriptive outcome data in the SOFA subgroups.

|  |  | Baseline SOFA <9 | |  | Baseline SOFA ≥9 | |
| --- | --- | --- | --- | --- | --- | --- |
|  |  | Usual protein | High protein |  | Usual protein | High protein |
| Characteristics | N | (N=315) | (N=311) | N | (N=338) | (N=333) |
| Age, years | 626 | 45/**59**/70 | 46/**59**/70 | 671 | 46/**59**/69.8 | 48/**59**/68 |
| Female | 626 | 42% 133/315 | 43% 133/311 | 670 | 40% 134/337 | 35% 116/333 |
| Admission category: |  |  |  |  |  |  |
| Medical | 626 | 82% 257/315 | 82% 256/311 | 671 | 83% 282/338 | 88% 292/333 |
| Surgical elective |  | 4% 12/315 | 5% 14/311 |  | 2% 7/338 | 3% 10/333 |
| Surgical emergency |  | 15% 46/315 | 13% 41/311 |  | 14% 49/338 | 9% 31/333 |
| COVID-19 positive on admission | 626 | 9% 29/315 | 8% 24/311 | 671 | 6% 19/338 | 4% 13/333 |
| BMI | 626 | 23/**27**/33 | 22/**26**/31 | 671 | 23/**26**/31.7 | 22/**26**/32 |
| Charlson Comorbidity Index | 626 | 0/**0**/1 | 0/**0**/1 | 671 | 0/**0**/2 | 0/**1**/2 |
| APACHE II score | 574 | 13/**17**/22 | 13/**18**/22 | 652 | 19/**24**/29 | 19/**24**/29 |
| mNUTRIC score | 574 | 2/**3**/5 | 2/**3**/5 | 652 | 4/**6**/7 | 4/**6**/7 |
| Frailty | 591 | 2/**3**/4 | 2/**3**/5 | 601 | 2/**3**/5 | 2/**3**/4 |
| SARC-F score | 581 | 0/**1**/4 | 0/**1**/5 | 583 | 0/**1**/5 | 0/**1**/4 |
| Renal replacement therapy on randomisation day | 626 | 8% 26/315 | 9% 28/311 | 671 | 21% 72/338 | 28% 93/333 |
| Acute kidney injury at time of randomisation*: | 626 |  |  | 671 |  |  |
| Yes |  | 17% 54/315 | 21% 64/311 |  | 28% 93/338 | 29% 98/333 |
| Stage 1 |  | 57% 31/54 | 45% 29/64 |  | 30% 28/93 | 30% 29/98 |
| Stage 2 |  | 22% 12/54 | 27% 17/64 |  | 20% 19/93 | 27% 26/98 |
| Stage 3 |  | 20% 11/54 | 28% 18/64 |  | 49% 46/93 | 44% 43/98 |
| Moderate or severe chronic renal disease^$^ | 626 | 4% 12/315 | 6% 19/311 | 671 | 12% 39/338 | 13% 44/333 |
| ICU length of stay | 621 | 5/**10**/20 | 6/**11**/19 | 670 | 5/**9**/18 | 5/**10**/18 |
| Hospital length of stay | 621 | 9/**19**/39 | 10/**21**/39 | 668 | 10/**19**/36 | 9/**18**/39 |
| 30-day mortality | 626 | 25% 78/315 | 22% 69/311 | 671 | 28% 93/338 | 34% 112/333 |
| 60-day mortality | 626 | 30% 94/315 | 27% 84/311 | 671 | 33% 113/338 | 41% 137/333 |

*a* ***b*** *c* represent the lower quartile *a*, the median *b*, and the upper quartile *c* for continuous variables. Categorical variables are described by counts and percentages. *N* is the number of non-missing values. Creatinine and urea are highest in 24 hours prior to randomisation. APACHE=Acute Physiology and Chronic Health Evaluation. ICU=intensive care unit. SOFA=Sequential Organ Failure Assessment. mNUTRIC=modified Nutrition Risk Assessment in Critical Illness Score. SARC-F= five item sarcopenia questionnaire score.

*Acute kidney injury refers to participants who met the criteria of KDIGO: stage 1 is at least 26.52 μmol/L increase in serum creatinine from baseline within 48 h or 1.5–1.9 times baseline within 7 days; stage 2 is 2.0–2.9 times baseline within 7 days; stage 3 is three times or more baseline within 7 days or increase to at least 353.6 μmol/L with an acute increase of more than 44.2 μmol/L.

^$^Defined in comorbidities as moderate [renal disease](https://www.sciencedirect.com/topics/medicine-and-dentistry/nephropathy): [creatinine clearance](https://www.sciencedirect.com/topics/medicine-and-dentistry/creatinine-clearance) 51–85 mL/min; and severe renal disease: creatinine clearance less than 50 mL/min and not on dialysis.

Table S7 Baseline characteristics and descriptive outcome data in the BMI subgroups.

|  |  | Baseline BMI ≤30 | |  | Baseline BMI >30 | |
| --- | --- | --- | --- | --- | --- | --- |
|  |  | Usual protein | High protein |  | Usual protein | High protein |
| Characteristics | N | (N=446) | (N=432) | N | (N=198) | (N=221) |
| Age, years | 878 | 46/**60**/70 | 42/**59**/70 | 419 | 47/**58**/66 | 48/**59**/69 |
| Female | 878 | 37% 165/446 | 36% 156/432 | 418 | 42% 84/198 | 50% 111/220 |
| Admission category: |  |  |  |  |  |  |
| Medical | 878 | 85% 377/446 | 81% 349/432 | 419 | 86% 171/198 | 86% 190/221 |
| Surgical elective |  | 4% 18/446 | 3% 13/432 |  | 3% 6/198 | 3% 6/221 |
| Surgical emergency |  | 11% 51/446 | 16% 70/432 |  | 11% 21/198 | 11% 25/221 |
| COVID-19 positive on admission | 878 | 4% 16/446 | 5% 22/432 | 419 | 11% 21/198 | 12% 26/221 |
| Charlson Comorbidity Index | 878 | 0/**0**/2 | 0/**0**/1 | 419 | 0/**1**/2 | 0/**0**/1 |
| SOFA | 878 | 6/**9**/11 | 6/**9**/11 | 419 | 7/**9**/12 | 6/**8**/11 |
| APACHE II score | 814 | 16/**21**/27 | 15/**21**/27 | 412 | 15/**20**/26 | 15/**21**/26 |
| mNUTRIC score | 814 | 3/**5**/6 | 3/**5**/6 | 412 | 3/**5**/6 | 3/**5**/6 |
| Frailty | 806 | 2/**3**/5 | 2/**3**/5 | 386 | 2/**3**/4 | 2/**3**/4 |
| SARC-F score | 792 | 0/**1**/5 | 0/**1**/4 | 372 | 0/**2**/5 | 0/**2**/4 |
| Renal replacement therapy on randomisation day | 878 | 16% 71/446 | 13% 56/432 | 419 | 21% 72/198 | 19% 42/221 |
| Acute kidney injury at time of randomisation*: | 878 |  |  | 419 |  |  |
| Yes |  | 24% 107/446 | 23% 99/432 |  | 35% 69/198 | 25% 55/221 |
| Stage 1 |  | 38% 38/100 | 40% 29/96 |  | 32% 20/62 | 41% 21/51 |
| Stage 2 |  | 28% 28/100 | 22% 17/96 |  | 24% 15/62 | 20% 10/51 |
| Stage 3 |  | 34% 34/100 | 39% 18/96 |  | 44% 27/62 | 39% 20/51 |
| Moderate or severe chronic renal disease^$^ | 878 | 9% 39/446 | 6% 28/432 | 419 | 12% 24/198 | 10% 23/221 |
| ICU length of stay | 876 | 6/**10**/19 | 5/**9**/18 | 415 | 6/**10**/17 | 5/**10**/24 |
| Hospital length of stay | 875 | 10/**21**/41 | 9/**19**/38 | 414 | 10/**18**/38 | 9/**19**/35 |
| 30-day mortality | 878 | 30% 133/446 | 28% 122/432 | 419 | 24% 48/198 | 22% 49/221 |
| 60-day mortality | 878 | 36% 159/446 | 34% 147/432 | 419 | 31% 62/198 | 27% 60/221 |

*a* ***b*** *c* represent the lower quartile *a*, the median *b*, and the upper quartile *c* for continuous variables. Categorical variables are described by counts and percentages. *N* is the number of non-missing values. Creatinine and urea are highest in 24 hours prior to randomisation. APACHE=Acute Physiology and Chronic Health Evaluation. ICU=intensive care unit. SOFA=Sequential Organ Failure Assessment. mNUTRIC=modified Nutrition Risk Assessment in Critical Illness Score. SARC-F= five item sarcopenia questionnaire score.

*Acute kidney injury refers to participants who met the criteria of KDIGO: stage 1 is at least 26.52 μmol/L increase in serum creatinine from baseline within 48 h or 1.5–1.9 times baseline within 7 days; stage 2 is 2.0–2.9 times baseline within 7 days; stage 3 is three times or more baseline within 7 days or increase to at least 353.6 μmol/L with an acute increase of more than 44.2 μmol/L.

^$^Defined in comorbidities as moderate [renal disease](https://www.sciencedirect.com/topics/medicine-and-dentistry/nephropathy): [creatinine clearance](https://www.sciencedirect.com/topics/medicine-and-dentistry/creatinine-clearance) 51–85 mL/min; and severe renal disease: creatinine clearance less than 50 mL/min and not on dialysis.

Figure S9 Heterogeneity of treatment effect analyses according to baseline BMI


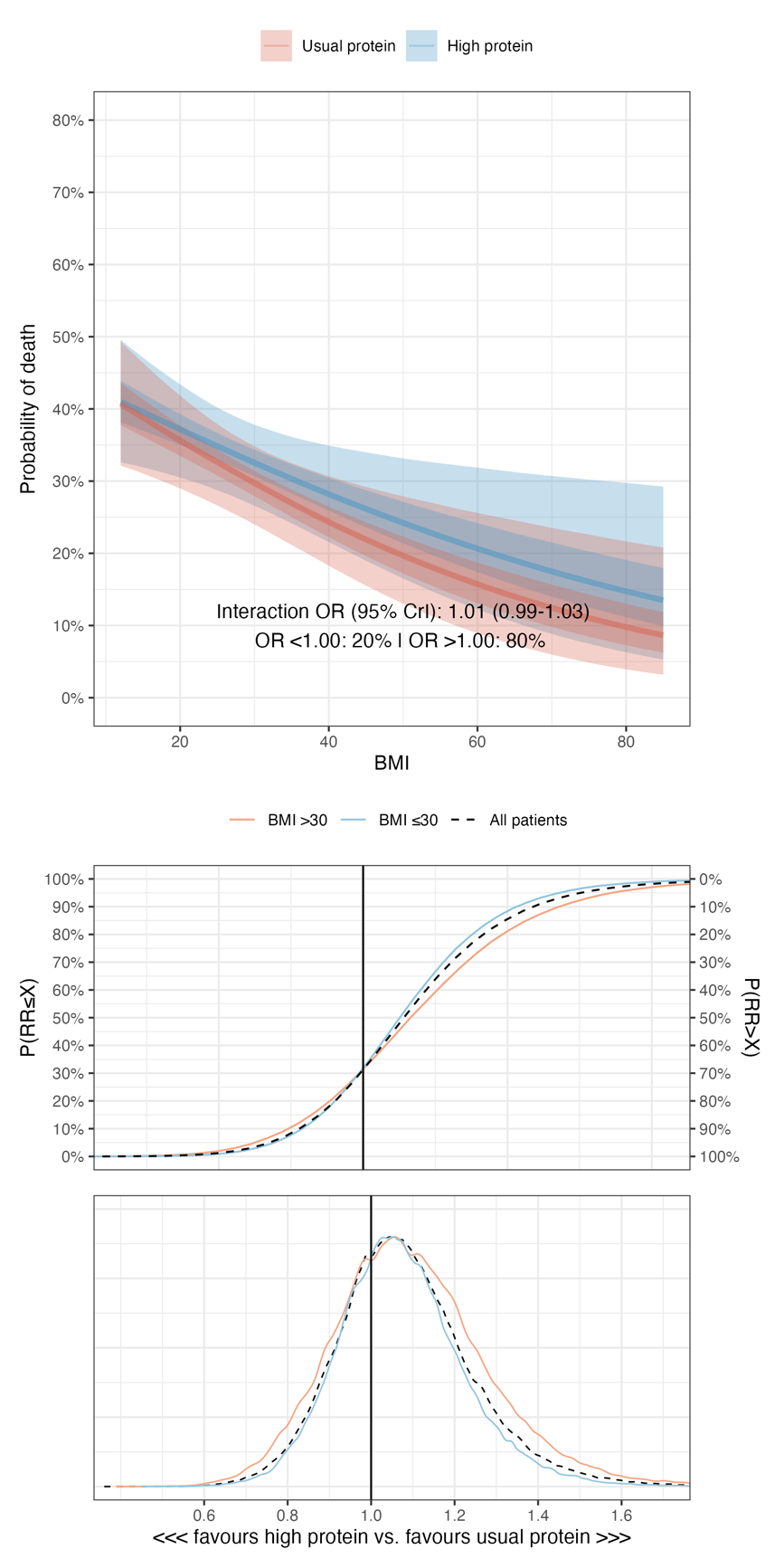


Analysis of heterogeneity of treatment effects for 60-day all-cause mortality using weakly informative priors.

Upper panel: conditional effects of the interaction between treatment allocation and baseline BMI (at randomisation). The plot displays the estimated mortality risk on the vertical axis and BMI on the horizontal axis. The conditional odds ratio of the interaction of treatment and BMI is 1.01 (95% credible interval: 0.99 to 1.03), and there is an 80% probability that mortality in the high protein group decreases with an increase of BMI. Lower panel: the cumulative posterior probability distributions with corresponding posterior density plots of the conditional risk ratios of 60-day all-cause mortality in the full sample and by BMI groups (BMI ≤30 and BMI >30).

**References**

1. Heyland DK, Patel J, Compher C, et al (2023) The effect of higher protein dosing in critically ill patients with high nutritional risk (EFFORT Protein): an international, multicentre, pragmatic, registry-based randomised trial. The Lancet 401:568–576. https://doi.org/10.1016/S0140-6736(22)02469-2

2. Sivapalan P, Meyhoff TS, Hjortrup PB, et al (2022) Conservative vs. liberal fluid therapy in septic shock – Protocol for secondary Bayesian analyses of the CLASSIC trial. Acta Anaesthesiol Scand 66:767–771. https://doi.org/10.1111/aas.14058

3. Sivapalan P, Meyhoff TS, Hjortrup PB, et al Restrictive versus standard IV fluid therapy in adult ICU patients with septic shock—Bayesian analyses of the CLASSIC trial. Acta Anaesthesiol Scand n/a: https://doi.org/10.1111/aas.14345

4. Andersen-Ranberg NC, Poulsen LM, Perner A, et al (2023) Haloperidol vs. placebo for the treatment of delirium in ICU patients: a pre-planned, secondary Bayesian analysis of the AID-ICU trial. Intensive Care Med 49:411–420. https://doi.org/10.1007/s00134-023-07024-9

5. Bürkner P-C (2017) brms: An R Package for Bayesian Multilevel Models Using Stan. J Stat Softw 80:1–28. https://doi.org/10.18637/jss.v080.i01

6. Zampieri FG, Casey JD, Shankar-Hari M, et al (2021) Using Bayesian Methods to Augment the Interpretation of Critical Care Trials. An Overview of Theory and Example Reanalysis of the Alveolar Recruitment for Acute Respiratory Distress Syndrome Trial. Am J Respir Crit Care Med 203:543–552. https://doi.org/10.1164/rccm.202006-2381CP

7. Statistical Rethinking: A Bayesian Course with Examples in R and STAN. In: Routledge CRC Press. https://www.routledge.com/Statistical-Rethinking-A-Bayesian-Course-with-Examples-in-R-and-STAN/McElreath/p/book/9780367139919. Accessed 12 Jan 2024

8. Carpenter B, Gelman A, Hoffman MD, et al (2017) Stan: A Probabilistic Programming Language. J Stat Softw 76:1–32. https://doi.org/10.18637/jss.v076.i01

9. Gelman A, Carlin JB, Stern HS, et al (2015) Bayesian Data Analysis, 3rd ed. Chapman and Hall/CRC, New York

**Appendix**

**A BAYESIAN reanalysis of the EFFORT trial: protocol**

Ryan W. Haines PhD, Zudin Puthucheary PhD, Andrew Day MD, John R. Prowle MD, Daren K. Heyland MD

Current Protocol Date: 13^th^ October 2023

Introduction

Establishing the optimal dose of protein or the effect of higher doses of protein supplementation has been identified as a research priority in the field of critical care nutrition to inform clinical practice guidelines. [(1)](https://www.zotero.org/google-docs/?Yykklk) Currently, a dose of 1.2 to 2.0 g/kg/day is recommended but based on weak evidence. [(1)](https://www.zotero.org/google-docs/?UY0mEt) There is no robust randomised controlled trial (RCT) evidence to guide protein delivery and conflicting evidence from observational studies.[(2)](https://www.zotero.org/google-docs/?g5VgzW) Observational evidence suggests higher doses of protein may improve outcomes possibly via better preservation of muscle function, improved physical recovery, and resultant reduced time to discharge. [(3,4)](https://www.zotero.org/google-docs/?REwILR) Conversely, adverse outcomes have been associated with high protein doses, with disrupted protein autophagy a proposed mechanism, leading to worse survival. [(5–7)](https://www.zotero.org/google-docs/?mDB0VM) The EFFORT trial aimed to address the uncertainty around optimal protein dose in critically ill patients with nutritional risk factors.

We outline a Bayesian secondary analysis of the EFFORT trial’s primary and secondary outcomes and analysis of heterogeneity of treatment effects (HTE). This will provide probabilities of effects of higher protein doses on outcomes. We will conduct post-hoc, secondary Bayesian analysis to provide a probabilistic interpretation of the primary outcome and further interrogate heterogeneity of treatment effects from the EFFORT-Protein results.

Methods

*Study design*

We performed this exploratory Bayesian analysis based on STROBE and proposed framework on Bayesian reanalysis of critical care trials.[(8)](https://www.zotero.org/google-docs/?AVuRQR) (<https://doi.org/10.1164/rccm.202006-2381CP>)

*The EFFORT trial*

A multicentre, pragmatic, volunteer-driven, registry-based, randomised, open-label, clinical trial comparing a higher protein dose (>= 2.2 g/kg/day) to a lower (<=1.2 g/kg/day) on time to discharge alive from hospital and 60-day mortality. The trial included 1329 adult (>= 18 years) participants with nutritional risk factors and requiring mechanical ventilation for >48 hours.

*Approvals*

The EFFORT trial is registered at clinicaltrials.gov (NCT03160547).

*COVID pandemic trial adjustments*

Due to the strain of the COVID-19 pandemic on critical care services and research prioritisation, the original enrolment goals were changed by the study steering committee on September 15, 2021 to end study enrolment at 1200 patients. This reduced the power for pre-planned frequentist analysis of the primary outcome of 60-day mortality. As a consequence, the secondary outcome of time to discharge alive (TTDA) was changed to the primary outcome due to realistic power to detect differences in effect size. This analysis will use the original primary outcome within the Bayesian framework.

*Statistical analysis*

We will use data from all patients in the modified intention-to-treat population of the EFFORT trial. All statistical analyses will be conducted using R (R Core Team, R Foundation for Statistical Computing) and Stan through the *brms* R package. We have used the same protocol and reporting structure as a recent Bayesian re-analyses in ICU [(9,10)](https://www.zotero.org/google-docs/?x0mIjK) and according to the principles outlined in HTE analyses.[(11)](https://www.zotero.org/google-docs/?ScKKmq)

*Descriptive data*

We will present descriptive baseline and outcome data as medians with interquartile ranges for numerical data, and as numbers with percentages for categorical data. A table of patient characteristics will be stratified by treatment allocation in the same format as the main publication. In addition, we will present variables included in the HTE analyses.

*Bayesian approach*

We will use Bayesian analyses to augment the interpretation of the EFFORT trial. We will use Bayesian methods to produce a distribution of effect sizes compatible with the trial data then interpreted in the context of pre-defined previous belief (‘priors’).

*Analysis of primary and secondary outcomes*

Primary outcome: 60-day mortality. We will explore the association of the primary endpoint and randomisation group by using a hierarchical Bayesian logistic regression model adjusted by intervention and trial site. We will use a family of priors (sceptical, optimistic, pessimistic), all assuming a normal distribution of the log odds ratio (log[OR]) and with a moderate uncertainty of the effect size.

EFFORT was initially designed to detect a between 4% absolute risk reduction from an absolute 30% mortality. This changed to 7.1% absolute risk reduction after the impact of COVID on trial recruitment and the planned sample size and therefore changed the primary outcome. However, for the purposes of prior definition in this analysis, we used the initial estimate to create more relevant priors. Considering a 30% base rate, we estimated a 4% reduction in absolute morality, or an odds ratio of ~0.82 (-0.198 on log scale). The optimistic prior was set as a normal prior on log scale with mean (-0.198) and standard deviation (0.195) set to detect 0.15 of probability of harm, fig 1A. The pessimistic prior was the opposite (mean 0.198, same standard deviation), to include a 0.15 probability of benefit, fig 1B. The neutral prior is set on an absence of effect (OR = 1; log[OR] = 0, standard deviation 0.355) and that 0.95 of all probability mass is contained between OR of 0.5 and 2.0, fig 1C.

A B C


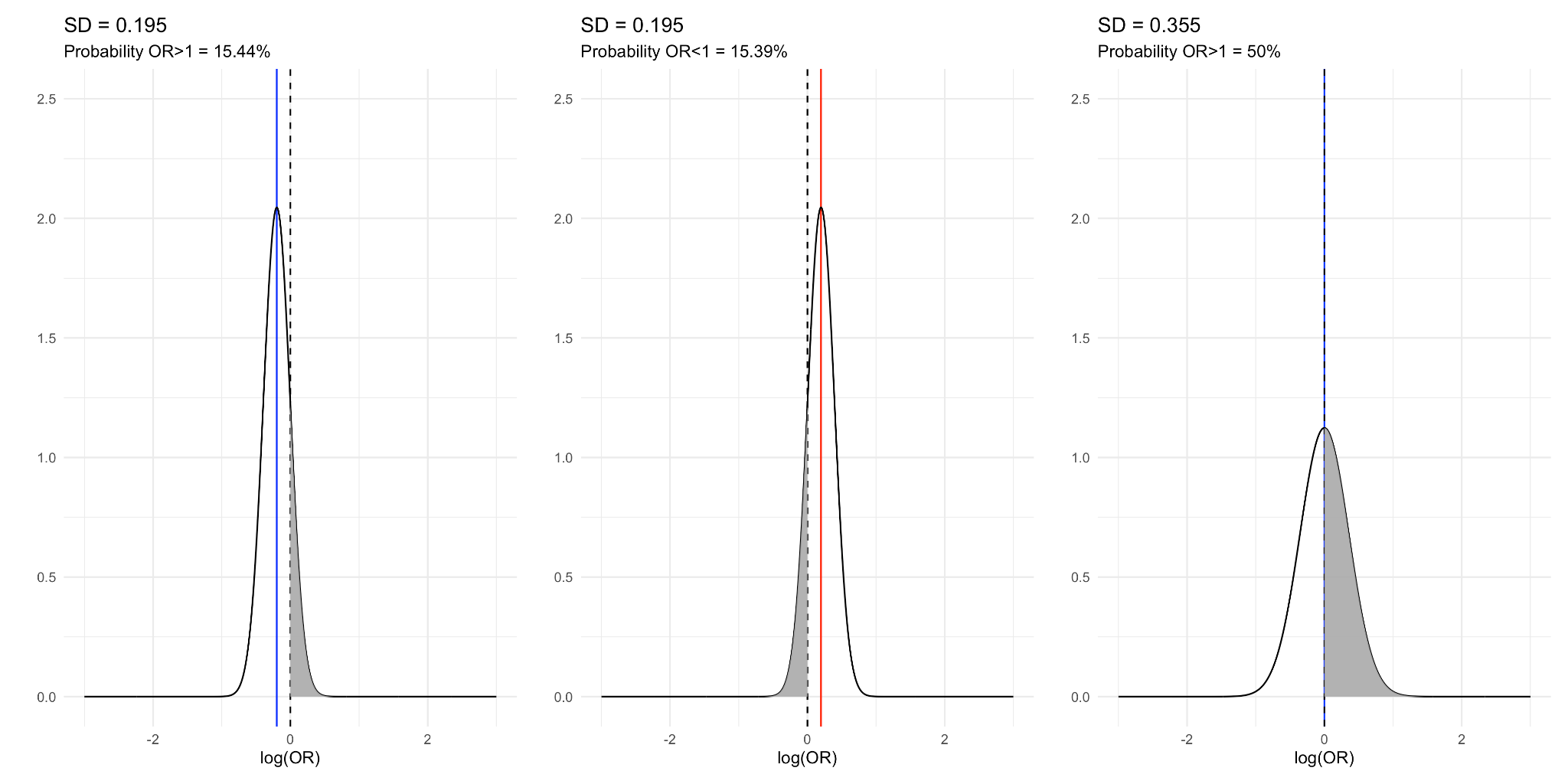


Figure 1. Optimistic (A), pessimistic (B), and sceptical (C) priors. OR - odds ratio. SD - standard deviation.

We will analyse TTDA from hospital using Bayesian regression with the “cox” family in brms an additional family of priors (sceptical, optimistic, pessimistic) with sceptical and optimistic centred on a 20% increase or decrease in hazard of TTDA, as outlined in the EFFORT trial power analysis. Hazard ratios of TTDA from hospital will be presented.

*HTE analysis in subgroups*

We will evaluate the presence of HTE for the primary outcome of 60-day mortality using Bayesian hierarchical logistic regression models. We will apply the approach outlined by Harrell [(12)](https://www.zotero.org/google-docs/?dSEV87) using formal interaction tests of pre-specified effect modifiers. [(13)](https://www.zotero.org/google-docs/?uwqj3U) Subgroups for interaction tests will include those outlined by EFFORT-Protein trial main manuscript:

- Acute kidney injury (AKI), AKI at enrolment, and creatinine value at baseline (on the continuous scale)
- Severity of illness (SOFA), at baseline (using both ≥9 cut point [(14)](https://www.zotero.org/google-docs/?v69ChM) and on the continuous scale)

Bayesian hierarchical logistic regression models partially pool data and shrink effects estimates of subgroups to the overall effect. [(9)](https://www.zotero.org/google-docs/?b6BW0j)

We will use the same neutral prior for the main effect. For interaction effects we will use weakly informative additional priors that apply regularisation and allow the likelihood to mostly determine the posterior (as outlined by Granholm et al [(9)](https://www.zotero.org/google-docs/?ZsbSi3):

- For the population-level intercept: a normally distributed prior with mean -0.4 and standard deviation (SD) 1.5. This corresponds to a prior baseline risk centred on 40% with 95% of probability mass between 3% and 93% for the control group.
- For group-level effects: a normally distributed prior with mean 0 and SD omega, with omega being the shrinkage factor and having a half-normally distributed prior with SD of 1.

We will use Bayesian logistic regression models to assess the interaction between Protein dose randomisation and SOFA score on the continuous scale. Results will be presented graphically as marginal effects plots for the interaction. We will use the following weakly informative priors:

- For the intercept and overall treatment effects, the same priors as specified for the subgroup analyses will be used.
- A normally distributed prior with mean 0 and SD of 0.1 for the interaction term of randomisation:SOFA.

All priors were chosen with the expectation the data from the EFFORT-Protein trial will dominate the posterior.

*Missing data handling*

We do not expect large numbers of missing data based on high levels of data completeness in previous trials [(15)](https://www.zotero.org/google-docs/?Yqe2S8). We will assess data missingness and if minimal (<1%) we will use mean imputation of baseline covariates [(16)](https://www.zotero.org/google-docs/?Rk61yt). Otherwise, we will perform multiple imputation with chained equations *mice* R package [(17)](https://www.zotero.org/google-docs/?KmHDHL).

Discussion

This Bayesian analysis will supplement the main trial findings. We will present the probabilities of the effect of higher protein doses on the original EFFORT study primary outcome. These analyses could produce results that allow clinicians to make clinically relevant statements about their updated beliefs from the data.

The analysis of HTE is well suited to Bayesian approaches where hierarchical models harness or learn from the effects of subgroups with large numbers to reduce the inherent volatility of smaller groups.

*Limitations*

These analyses remain secondary to the main analysis of the trial and have several limitations. The Bayesian approach will not eliminate biases from the RCT including change in sample size due to COVID-19 and open-label design. Interpretation of HTE is only hypothesis generating and despite this analysis plan, is at risk of bias due to smaller numbers of patients when analysing sub-groups.

Conclusion

The Bayesian framework will help clinicians and researchers understand the effect of higher doses of protein on critically ill patients by presenting direct evidence of clinical benefit or harm.

References

[1. Compher C, Bingham AL, McCall M, Patel J, Rice TW, Braunschweig C, et al. Guidelines for the provision of nutrition support therapy in the adult critically ill patient: The American Society for Parenteral and Enteral Nutrition. J Parenter Enter Nutr. 2022;46(1):12–41.](https://www.zotero.org/google-docs/?GD9wv1)

[2. Lee ZY, Yap CSL, Hasan MS, Engkasan JP, Barakatun-Nisak MY, Day AG, et al. The effect of higher versus lower protein delivery in critically ill patients: a systematic review and meta-analysis of randomized controlled trials. Crit Care. 2021 Jul 23;25(1):260.](https://www.zotero.org/google-docs/?GD9wv1)

[3. Weijs PJM, Looijaard WGPM, Beishuizen A, Girbes ARJ, Oudemans-van Straaten HM. Early high protein intake is associated with low mortality and energy overfeeding with high mortality in non-septic mechanically ventilated critically ill patients. Crit Care Lond Engl. 2014 Dec 14;18(6):701.](https://www.zotero.org/google-docs/?GD9wv1)

[4. Allingstrup MJ, Esmailzadeh N, Wilkens Knudsen A, Espersen K, Hartvig Jensen T, Wiis J, et al. Provision of protein and energy in relation to measured requirements in intensive care patients. Clin Nutr Edinb Scotl. 2012 Aug;31(4):462–8.](https://www.zotero.org/google-docs/?GD9wv1)

[5. Casaer MP, Mesotten D, Hermans G, Wouters PJ, Schetz M, Meyfroidt G, et al. Early versus Late Parenteral Nutrition in Critically Ill Adults. N Engl J Med. 2011 Aug 11;365(6):506–17.](https://www.zotero.org/google-docs/?GD9wv1)

[6. Casaer MP, Wilmer A, Hermans G, Wouters PJ, Mesotten D, Van den Berghe G. Role of Disease and Macronutrient Dose in the Randomized Controlled EPaNIC Trial. Am J Respir Crit Care Med. 2013 Feb;187(3):247–55.](https://www.zotero.org/google-docs/?GD9wv1)

[7. Puthucheary ZA, Rawal J, McPhail M, Connolly B, Ratnayake G, Chan P, et al. Acute skeletal muscle wasting in critical illness. JAMA. 2013 Oct 16;310(15):1591–600.](https://www.zotero.org/google-docs/?GD9wv1)

[8. Zampieri FG, Casey JD, Shankar-Hari M, Harrell FE, Harhay MO. Using Bayesian Methods to Augment the Interpretation of Critical Care Trials. An Overview of Theory and Example Reanalysis of the Alveolar Recruitment for Acute Respiratory Distress Syndrome Trial. Am J Respir Crit Care Med. 2021 Mar 1;203(5):543–52.](https://www.zotero.org/google-docs/?GD9wv1)

[9. Granholm A, Marker S, Krag M, Zampieri FG, Thorsen-Meyer HC, Kaas-Hansen BS, et al. Heterogeneity of treatment effect of stress ulcer prophylaxis in ICU patients: A secondary analysis protocol. Acta Anaesthesiol Scand. 2019;63(9):1251–6.](https://www.zotero.org/google-docs/?GD9wv1)

[10. Sivapalan P, Meyhoff TS, Hjortrup PB, Lange T, Møller MH, Perner A, et al. Conservative vs. liberal fluid therapy in septic shock – Protocol for secondary Bayesian analyses of the CLASSIC trial. Acta Anaesthesiol Scand. 2022;66(6):767–71.](https://www.zotero.org/google-docs/?GD9wv1)

[11. Iwashyna TJ, Burke JF, Sussman JB, Prescott HC, Hayward RA, Angus DC. Implications of Heterogeneity of Treatment Effect for Reporting and Analysis of Randomized Trials in Critical Care. Am J Respir Crit Care Med. 2015 Nov 1;192(9):1045–51.](https://www.zotero.org/google-docs/?GD9wv1)

[12. Jr FEH. Biostatistics for Biomedical Research [Internet]. 2022 [cited 2022 Jul 13]. Available from: https://hbiostat.org/bbr/](https://www.zotero.org/google-docs/?GD9wv1)

[13. Hoogland J, IntHout J, Belias M, Rovers MM, Riley RD, E. Harrell Jr F, et al. A tutorial on individualized treatment effect prediction from randomized trials with a binary endpoint. Stat Med. 2021;40(26):5961–81.](https://www.zotero.org/google-docs/?GD9wv1)

[14. Heyland DK, Patel J, Compher C, Rice TW, Bear DE, Lee ZY, et al. The effect of higher protein dosing in critically ill patients with high nutritional risk (EFFORT Protein): an international, multicentre, pragmatic, registry-based randomised trial. The Lancet. 2023 Feb 18;401(10376):568–76.](https://www.zotero.org/google-docs/?GD9wv1)

[15. Heyland D, Muscedere J, Wischmeyer PE, Cook D, Jones G, Albert M, et al. A randomized trial of glutamine and antioxidants in critically ill patients. N Engl J Med. 2013 Apr 18;368(16):1489–97.](https://www.zotero.org/google-docs/?GD9wv1)

[16. White IR, Thompson SG. Adjusting for partially missing baseline measurements in randomized trials. Stat Med. 2005;24(7):993–1007.](https://www.zotero.org/google-docs/?GD9wv1)

[17. Buuren S van, Groothuis-Oudshoorn K. mice: Multivariate Imputation by Chained Equations in R. J Stat Softw. 2011 Dec 12;45:1–67.](https://www.zotero.org/google-docs/?GD9wv1)
