# Supplementary material for: The effect of high protein dosing in critically ill patients: an exploratory, secondary Bayesian analyses of the EFFORT Protein trial
Source: Br J Anaesth. 2024 Oct 24;133(6):1192–200. doi: 10.1016/j.bja.2024.08.033 (PMC11589476; doi:10.1016/j.bja.2024.08.033)
Supplement: Multimedia component 3 [file mmc3.docx]

**Instrument to assess the credibility of effect modification analyses (ICEMAN)**

**in a randomized controlled trial**

*Version 1.1*

**Consider the following important instructions informed by common misapplications of ICEMAN in studies using the instrument**

- Complete a separate credibility assessment per each effect modifier (e.g., age, comorbidity, drug dose, etc.), outcome (e.g., mortality, stroke, duration of hospital stay), time-point (e.g., 3 months, 6 months), and effect measure (e.g. relative risk, risk difference).
- Do not apply ICEMAN if the interaction p-value is 0.1 or larger, i.e., provides very little statistical support for the existence of an effect modification (ICEMAN is designed to address the possible claim of an effect modification rather than the claim of no effect modification).
- Response options on the left indicate definitely or probably reduced credibility, response options on the right probably or definitely increased credibility
- Completely unclear should be interpreted as probably reduced credibility.
- To ensure transparency, provide a supporting comment under each question that provides a rationale for the rating.
- To ensure transparency, provide a copy of the completed ICEMAN instrument in the supplement of your article.

| **CREDIBILITY ASSESSMENT** | | | |
| --- | --- | --- | --- |
| **Essential preliminary considerations to define the possible effect modification of interest** | | | |
| State a single candidate effect modifier (e.g., age or comorbidity): BMI | | | |
| Was the effect modifier measured before or at randomization? Yes, continue [ ] no, stop here and refer to manual for further instructions | | | |
| State a single outcome and time-point (e.g., mortality at 1 year follow-up): 60-day mortality | | | |
| State a single effect measure (e.g., relative risk or risk difference): OR | | | |
| **1: Was the direction of the effect modification correctly hypothesized a priori?** | | | |
| Definitely no | [ ] Probably no or unclear | [ ] Probably yes | [ ] Definitely yes |
| *Clearly post-hoc or results inconsistent with hypothesized direction or biologically very implausible* | *Vague hypothesis or hypothesized direction unclear* | *No prior protocol available but unequivocal statement of a priori hypothesis with correct direction of effect modification* | *Prior protocol available and includes correct specification of direction of effect modification, e.g. based on a biologic rationale* |
| Comment: | | | |
| **2: Was the effect modification supported by prior evidence?** | | | |
| [ ] Inconsistent with prior evidence | Little or no support or unclear | [ ] Some support | [ ] Strong support |
| *Prior evidence suggested a different direction of effect modification* | *No prior evidence or consistent with weak or very indirect prior evidence (e.g. animal study at high risk of bias) or unclear* | *Consistent with more limited or indirect prior evidence (e.g. large observational study, non-significant effect modification in prior RCT, or different population)* | *Consistent with strong prior evidence directly applicable to the clinical scenario (e.g. significant effect modification in related RCT)* |
| Comment: | | | |
| **3: Does a test for interaction suggest that chance is an unlikely explanation of the apparent effect modification?** (consider irrespective of number of effect modifiers) | | | |
| [ ] Chance a very likely explanation | Chance a likely explanation or unclear | [ ] Chance may not explain | [ ] Chance an unlikely explanation |
| *Interaction p-value >0.05* | *Interaction p-value ≤0.05 and >0.01, or no test of interaction reported and not computable* | *Interaction p-value ≤0.01 and >0.005* | *Interaction p-value ≤0.005* |
| Comment: | | | |
| **4: Did the authors test only a small number of effect modifiers or consider the number in their statistical analysis?** | | | |
| [ ] Definitely no | [ ] Probably no or unclear | [ ] Probably yes | Definitely yes |
| *Explicitly exploratory analysis or large number of effect modifiers tested (e.g. greater than 10) and multiplicity not considered in analysis* | *No mention of number or 4-10 effect modifiers tested and number not considered in analysis* | *No protocol available but unequivocal statement of 3 or fewer effect modifiers tested* | *Protocol available and 3 or fewer effect modifiers tested or number considered in analysis* |
| Comment: | | | |
| **5: If the effect modifier is a continuous variable, were arbitrary cut points avoided?** [ ] not applicable: not continuous | | | |
| Definitely no | [ ] Probably no or unclear | [ ] Probably yes | [ ] Definitely yes |
| *Analysis based on exploratory cut point (e.g. picking cut point associated with highest interaction p-value)* | *Analysis based on cut point(s) of unclear origin* | *Analysis based on pre-specified cut points, e.g. suggested by prior RCT* | *Analysis based on the full continuum, e.g. assuming a linear or logarithmic relationship* |
| Comment: | | | |
| **6 Optional: Are there any additional considerations that may increase or decrease credibility?** (manual section 2.6) | | | |
|  | [ ] yes, probably decrease | [ ] yes, probably increase | |
| Comment:   \| **7: How would you rate the overall credibility of the proposed effect modification?**  The overall rating should be driven by the items that decrease credibility. The following provides a sensible strategy:   - All responses definitely or probably reduced credibility or unclear 🡪 very low - Two or more responses definitely reduced credibility 🡪 maximum usually low even if all other responses satisfy credibility criteria - One response definitely reduced credibility 🡪 maximum usually moderate even if all other responses satisfy credibility criteria - Two responses probably reduced credibility 🡪 maximum usually moderate even if all other responses satisfy credibility criteria - No response options definitely or probably reduced credibility 🡪 high very likely   Place a mark on the continuous line (e.g. hit “x” in electronic version) \| \| \| \| \|  \| \| --- \| --- \| --- \| --- \| --- \| --- \| \|  \|  \| \| \| \|  \| \|  \| **X** \| \| \| \|  \| \|  \|  \| \|  \|  \| \| \| \|  \| \|  \|  \| \| \| \|  \| \|  \| **Very low credibility** \| **Low credibility** \| **Moderate credibility** \| **High credibility** \|  \| \|  \|  \|  \|  \|  \|  \| \|  \| Minimal to no support for effect modification;  Use overall effect for each subgroup \| Some but insufficient support for effect modification;  Use overall effect for each subgroup but note remaining uncertainty \| Likely effect modification;  Use separate effects for each subgroup but note remaining uncertainty \| Very likely effect modification;  Use separate effects for each subgroup \|  \| \| Comment: \| \| \| \| \| \| | | | |

# **Limited evidence for effect nmodification of BMI on treatment**
